# A Comprehensive Analysis of Dietary Fishmeal Replaced by Black Soldier Fly (*Hermetia illucens* L.) in Largemouth Bass (*Micropterus salmoides*)

**DOI:** 10.1155/anu/7583815

**Published:** 2026-06-25

**Authors:** Yang Chen, Xirui Li, Xinyu Li, Hang Su, Xiangyu She, Ruyi Li, Kangsen Mai, Fei Song

**Affiliations:** ^1^ Institute of Modern Aquaculture Science and Engineering (IMASE), Guangdong Provincial Key Laboratory of Insect Developmental Biology and Applied Technology, Institute of Insect Science and Technology and School of Life Sciences, South China Normal University, Guangzhou, China, scnu.edu.cn; ^2^ Guangdong Yuehai Feed Group Co., Ltd., Zhanjiang, China; ^3^ Key Laboratory of Aquaculture Nutrition (Ministry of Agriculture and Rural Affairs), Ocean University of China, Qingdao, China, ouc.edu.cn; ^4^ Southern Marine Science and Engineering Guangdong Laboratory (Zhuhai), Zhuhai, China, sysu.edu.cn

**Keywords:** aquafeeds, BFLM, fatty acids composition, improved efficiency, substitution

## Abstract

Black soldier fly larvae meal (BFLM) is characterized by high yield and a well‐balanced nutritional profile, making it a promising alternative protein source for aquafeeds in the near further. Previous studies have demonstrated its potential to partially replace fishmeal (FM) in aquatic diets. Therefore, a 56‐day feeding trial was conducted in largemouth bass (LMB) to evaluate the effects of graded level of BFLM (0%, 17%, 34%, 51%, and 68%) as a substitute for FM on growth performance, antioxidant, immune responses, and muscle quality. The results showed that dietary BFLM replacement levels above 17% significantly reduced SGR, FER, and whole‐body crude lipid content, thereby impairing growth performance. In addition, higher substitution levels disrupted the antioxidant system, promoted the expression of inflammatory cytokines through activation of the nrf2‐keap1 pathway, and consequently induced inflammatory responses. Furthermore, BFLM replacement exceeding 17% decreased muscle hardness and chewiness, reduced the content of aroma‐, umami‐, and sweet‐related compounds, and increased the levels of off‐flavor substances and SFAs, ultimately leading to deterioration in muscle quality. Based on the present results, the optimal replacement level of BFLM in diets for LMB should not exceed 17%. A comprehensive analysis of the adverse effects associated with high levels of BFLM substitution indicated that the high content of saturated fatty acids (FAs) and the low content of long‐chain polyunsaturated FAs in BFLM were the key factors limiting growth performance, inducing oxidative stress, and reducing muscle quality. To improve the efficiency of BFLM as a substitute for FM, it is necessary to optimize FA composition while comprehensively considering growth performance, antioxidant and immune status, and muscle quality.

## 1. Introduction

Aquaculture has become an increasingly important contributor to the global supply of animal‐derived protein and will continue to play a critical role in the future [1]. Global aquafeed production is predicted to increase by 75% between 2015 and 2025. In 2024, aquaculture production exceeded capture fisheries production for the first time. Notably, aquafeeds are a key pillar to supporting the development of the aquaculture industry [2]. These increases have been largely driven by the stable supply of feed ingredients [3, 4]. Therefore, identifying cost‐effective and high‐quality protein sources is essential to meet the demands of the continuous and sustainable development of aquaculture.

In recent years, increasing numbers of studies have suggested that insect protein has great potential as a high‐quality protein source for aquafeeds compared with conventional protein sources [5]. Insects generally have short life cycles, are easy to cultivate, and exhibit high productivity and feed conversion efficiency. Moreover, insect proteins possess a balanced nutritional composition, which is crucial for aquatic animals [6]. In addition, the search for new protein sources for aquafeeds should also take into account the reduction of carbon footprints [7, 8]. Insects can utilize food waste as a nutrient source for biotransformation, thereby producing valuable protein ingredients, and are therefore regarded as one of the most promising novel protein sources for animal production. Among the insect species currently receiving attention, the black soldier fly (*Hermetia illucens* L.) is considered one of the most promising candidates [9]. To date, many studies have evaluated its feasibility as a substitute for fishmeal (FM) in a wide range of aquatic animals. Studies in both fish and crustacean species have demonstrated the potential of black soldier fly larvae meal (BFLM) to replace FM [10, 11]. However, these studies have also shown that the effects of BFLM on growth performance vary considerably depending on fish species and the source of BFLM. Accordingly, the optimal substitution level of BFLM for FM has been reported to range widely from less than 10%–70% [12, 13]. Furthermore, many previous studies have focused primarily on the growth performance, which limits a comprehensive evaluation of the true substitution efficiency of BFLM. Therefore, a more systematic and integrated assessment of BFLM as an alternative protein source is needed.

Nowadays, the aquaculture industry is placing increasing emphasis on the health status and product quality of farmed animals [14]. This trend is driven not only by market demand but also by the requirements of sustainable development. Therefore, in addition to growth performance, it is also necessary to systematically evaluate stress resistance and muscle quality when assessing the efficiency of novel protein sources. In the present study, largemouth bass (LMB; *Micropterus salmoides*), a fast‐growing species recognized for its high nutritional value and strong environmental adaptability, was selected as the experimental model [15, 16]. Previous studies have investigated the effects of BFLM in LMB diets on growth performance [17], immune responses [18], and fillet quality [19], respectively, However, the comprehensive effects of replacing dietary FM with BFLM on growth, stress resistance, and muscle quality have scarcely been reported. Therefore, the present study was conducted to clarify the mechanisms underlying growth inhibition, oxidative stress, and muscle quality deterioration induced by high levels of BFLM and to define the appropriate inclusion level of BFLM in practical diets for LMB.

## 2. Materials and Methods

### 2.1. Feed Preparation and Fish Management

Procedures used in this study strictly complied with the regulations of the University Animal Care and Use Committee of South China Normal University (the approval reference number 1002019–02‐0016).

The composition of the experimental diets is given in Table 1. FM was replaced by BFLM at levels of 0%, 17%, 34%, 51%, and 68%, respectively. The BFLM used in this experiment was obtained from larvae reared to the pre‐pupal stage. After repeated rinsing with clean water to remove impurities, the larvae were inactivated in a boiling water bath at 100°C for 5 min. The inactivated larvae were then dried to constant weight in a hot‐air oven at 65°C, ground using a high‐speed grinder, and passed through a 60‐mesh sieve to obtain black soldier fly meal. The resulting meal was stored in sealed bags at 4°C until use. The fatty acid (FA) and amino acid (AA) compositions of the feed ingredients are presented in Tables S1 and S2. All dietary ingredients were well ground into powder through a 200 μm screen and finely mixed with fish oil and soybean oil according to the designed formulations. The dough was pressed into pellets using a pelletizer (F‐26, South China University of Technology, Guangzhou, China). After that, all diets were air‐dried at 45°C for 24 h and then stored at −20°C until use [20].

**Table 1:** Experimental formulation design and nutritional composition (%, dry weight).

Ingredients	Diet groups				
	BFLM0	BFLM17	BFLM34	BFLM51	BFLM68
Fishmeal^a^	25.00	20.75	16.50	12.25	8.00
Black soldier fly larvae meal^b^	0.00	7.50	15.00	22.50	30.00
Poultry by‐product meal^c^	20.00	20.00	20.00	20.00	20.00
Poultry blood meal	16.00	16.00	16.00	16.00	16.00
Cottonseed protein	5.00	5.00	5.00	5.00	5.00
Gluten powder	2.50	2.50	2.50	2.50	2.50
Hydrolyzed feather meal	1.50	1.50	1.50	1.50	1.50
Starch	9.00	9.00	9.00	9.00	9.00
Soybean oil	8.00	6.00	4.00	2.00	0.00
Soybean lecithin	2.00	2.00	2.00	2.00	2.00
Ca(H_2_PO_4_)_2_	1.00	1.00	1.00	1.00	1.00
Choline chloride	0.40	0.40	0.40	0.40	0.40
Vitamin premix^d^	1.50	1.50	1.50	1.50	1.50
L‐lysine‐HCL	0.00	0.02	0.05	0.07	0.10
DL‐methionine	0.26	0.29	0.32	0.34	0.37
L‐threonine	0.14	0.15	0.15	0.16	0.16
Mineral premix^e^	0.50	0.50	0.50	0.50	0.50
Ethoxyquin	0.01	0.01	0.01	0.01	0.01
Propionic acid	0.07	0.07	0.07	0.07	0.07
Cellulose	7.12	5.81	4.50	3.20	1.89
Total	100.00	100.00	100.00	100.00	100.00
Proximate composition (%)					
Crude protein	51.39	51.40	51.41	51.42	51.43
Crude lipid	14.71	14.75	14.79	14.83	14.87

^a^Fishmeal was sourced from Rongcheng Haihesheng Marine Biotechnology Co., Ltd.

^b^Black soldier fly larvae meal was sourced from Biosource Biotechnology (Shenzhen) Co., Ltd.

^c^Poultry by‐product meal was sourced from Fujian Haisheng Feed Co., Ltd.

^d^Vitamin premix (mg/kg): nicotinamide, 79.20; calcium pantothenate, 73.60; folic acid, 6.40; biotin, 0.64; inositol, 320; L, carnitine; 100; vitamin A, 16,000 IU; vitamin D3, 8000 IU; vitamin B1, 17.80; vitamin B2, 48.00; vitamin B6, 29.52; vitamin B12, 0.24; vitamin C, 800; vitamin K3, 14.72; vitamin E, 160.

^e^Mineral premix (mg/kg): Mg (MgSO_4_ · H_2_O), 52.70; Zn (ZnSO_4_), 34.40; Fe (FeSO_4_), 21.10; Mn (MnSO_4_), 6.20; Cu (CuSO_4_), 2.00; (Ca (IO_3_)_2_), 1.63; Se (Na_2_SeO_3_), 0.18; Co (COCl_2_), 0.24.

Juvenile LMB were obtained from the Hualai Fish Sprout Factory (Foshan, China). Fish were fed to satiety with a commercial feed twice daily under a natural photoperiod. After an acclimatization period of 4 weeks to laboratory conditions, 400 LMB, with a mean initial weight of 26.00 ± 0.01 g, were randomly distributed into 20 fish tanks, resulting in 20 juveniles in each tank. No confounding factors were controlled for. Each experimental diet was randomly assigned to tanks in quadruplicate. After the experiment began, LMB were fed twice a day at 8:00 and 17:00. During the experimental period, water temperature, NH_4_‐N and dissolved oxygen were 28 ± 2°C, 84 ± 16 μg/L, and 6.5 ± 0.5 mg/L, respectively.

### 2.2. Sample Collection and Analysis

After the 8‐week experiment, all fish were starved for 24 h. LMB from each tank were counted and weighed. Six fish were randomly selected from each tank: 2 for whole‐body nutrient composition analysis and the remaining four were anesthetized with MS‐222 (100 mg/L) for tissue collection. For these four fish, muscle was divided into two parts: molecular samples for subsequent molecular analysis and crude composition samples for nutrient analysis; liver, intestine, and head kidney were rapidly dissected, weighed, and snap‐frozen in liquid nitrogen. Blood was collected from the caudal vein into EDTA‐anticoagulated vacutainers, kept on ice, and centrifuged (3000 × *g*, 10 min, 4°C) to obtain plasma. All samples were stored at −80°C until further analysis [20].

During the culture period, feed intake and fish mortality were recorded daily. The following parameters were calculated based on the sampling data:

Weight gain rate WGR,%=100×Wf−Wi/Wi,

Specific growth rate SGR,%/day=100×lnWf− lnWi/t,

Survival rate SR,%=100×Nf/Ni,

Feed efficiency ratio FER=Wf−Wi/Dfeed,

Condition factor CF, g/cm3=100×Wf/L3,

Viscerosomatic index VSI,%=100×Wv/Wf,

Hepatosomatic index HSI,%=100×Wh/Wf,

Relative gut length RGL,%=100×Lg/L,

where *W*_i_ is the initial body weight (g); *W*_f_ is the final body weight (g); *N*_i_ is the initial number of fish; *N*_f_ is the final number of fish; *t* is the experimental duration (days); *D*_feed_ is the dry feed intake (g); *W*_v_ is the viscerosomatic weight (g); *W*_h_ is the hepatosomatic weight (g); *L*_g_ is the gut length (cm); and *L* is the body length (cm).

### 2.3. Nutritional Composition Analysis of Whole Fish and Muscle

Moisture content was conducted by drying samples to a constant weight in an oven at 105°C and calculating it as a percentage. The crude lipid content was measured by a petroleum ether extractor (B.P. 30–60°C, 3 h) in a Soxtec 2055 extraction. Crude protein content was analyzed using a Dumas nitrogen determination apparatus (DT autosampler, Europe Gerhardt Company, Königswinter, Germany). Free AA (FAA) and bound AA composition in the muscle were determined using an automatic AA analyzer (LA8080; Hitachi, Tokyo, Japan). FA composition was measured following the method of Xu et al. [21]. For plasma AA analysis, 400 μL of plasma was deproteinized with 1.2 mL of 10% sulfosalicylic acid (SSA), incubated at room temperature for 5 min, and centrifuged at 13,000 × *g* for 15 min at 4°C. The supernatant was then filtered through a 0.22 μm membrane prior to analysis. For muscle samples, 0.2 g of tissue was homogenized in 1.2 mL of 10% SSA using a cryogenic grinder (70 Hz, 15 s × 6 cycles, with cooling intervals) in the presence of zirconium beads. After centrifugation at 13,000 × *g* for 15 min at 4°C, the supernatants were filtered through a 0.22 μm membrane. The hydroxyproline (H‐pro) content was determined using a commercial kit (A030–2–1; Jiancheng Bioengineering Institute, Nanjing, China) according to the manufacturer’s instructions based on the alkaline hydrolysis method. The collagen content was determined according to the method of Crouse et al. [22]. Since hydroxyproline accounts for 13.4% of collagen, collagen content was calculated accordingly.

### 2.4. Plasma and Tissue Biochemical Indicators Assay

Plasma samples stored at −80°C were thawed on ice prior to the analysis to minimize protein degradation. A volume of 200 μL plasma was collected for biochemical measurements. Concentrations of total protein (TP), total cholesterol (CHOL), triglycerides (TG), glucose (GLU), high‐density lipoprotein (HDL) CHOL, and low‐density lipoprotein (LDL) CHOL were measured using a fully automated biochemical analyzer (Hitachi CHEMIX‐800, Sysmex Corporation, Kobe, Japan). The activities or concentrations of antioxidant‐related indicators and metabolites in muscle and liver, including β‐alanine, carnosine (CAR), anserine (ANS), CAR synthase 1 (CARNS1), CAR N‐methyltransferase (CNMT), total antioxidant capacity (T‐AOC), malondialdehyde (MDA), reactive oxygen species (ROS), superoxide dismutase (SOD), catalase (CAT), glutathione (GSH), GSH S‐transferase (GST), NAD(P)H quinone oxidoreductase 1 (NQO1), and heme oxygenase‐1 (HO‐1), were measured using commercially available kits purchased from Jiangsu Meimian Industrial Co., Ltd. and Guangzhou Aoruida Biotechnology Co., Ltd.

### 2.5. Oil Red O‐Stained Liver Sections and TG Measurement

Oil red O‐stained liver sections prepared by Guangzhou Servicebio were observed and imaged using a Leica DM 6000 microscope. The lipid droplet area (%) was quantified using ImageJ software. Concurrently, hepatic triglyceride levels were quantified using a GPO‐PAP assay kit (A110–1–1, Nanjing Jiancheng, China). The liver tissue was homogenized in an appropriate volume of PBS using a freezing grinder until complete homogenization, followed by centrifugation at 3000 × *g* for 10 min at 4°C. The TG content was normalized to the tissue protein concentration (μmol/g protein).

### 2.6. RNA Extraction and Real‐Time Quantitative PCR

Total RNA was isolated with TRIzol reagent (Vazyme Biotech Co, China) according to the manufacturer’s instructions. After elution in DEPC‐treated water, RNA integrity and concentration were verified. One microgram of total RNA was used for cDNA synthesis with the PrimeScript RT reagent Kit (R223−01; Vazyme, Nanjing, China). Quantitative PCR was performed on a CFX96 real‐time PCR system (Bio‐Rad, Laboratories, Inc.). Two reference genes (β‐actin and EF1α) were evaluated, and both were used for normalization. Their expression stability was validated via geNorm and NormFinder software. Prior to q‐PCR, amplification efficiencies of target and reference genes were assessed by standard curves (log cDNA dilution vs. ΔCT) and incorporated into the analysis. Efficiency (*E*) was calculated as *E* = 10^−1/slope^ − 1, with reliable *E* (0.9–1.1) corresponding to slopes of −3.6 to −3.1 (Vazyme instructions). Relative gene expression was calculated using the 2^−ΔΔCt^ method. Primers are shown in Table S3, and q‐PCR procedures followed our previous study [23].

### 2.7. Muscle Texture Assay

Two fish were randomly selected from each tank, and dorsal muscle samples were collected from the same side of each fish. A rectangular muscle sample (1 cm × cm1 × 0.5 cm) was gently obtained using a surgical blade and analyzed within 24 h. Texture profile analysis (TPA) was performed using a TA.XT Plus texture analyzer (Stable Micro Systems, UK) equipped with a flat‐bottom cylindrical probe. Each fresh sample was compressed twice under the following settings: pre‐test speed 5 mm/s, test speed 1 mm/s, compression 60%, 5 s interval, and data collection rate 5 mm/s.

### 2.8. Muscle Volatile Assay

Muscle volatile compounds were determined using the method described by Zhang et al. [24] with modifications. A total of 100 ± 1 mg of the sample was placed in a 70 Hz grinder for 30 s. Subsequently, 500 μL of pre‐cooled extract (methanol:isopropanol:water = 3:3:2, v/v/v) was added and vortex‐mixed for 3 min followed by 20 min of ultrasound in an ice‐water bath. The sample was centrifuged at 4°C and 12,000 rpm for 10 min. To 300 μL of the supernatant, 20 μL of internal standard (10 μg/mL) was added. The mixture was then dried under a nitrogen stream. After lyophilization in a freeze dryer, 100 μL of methoxyamination hydrochloride (15 mg/mL in pyridine) was added, and the mixture was incubated at 37°C for 2 h, then derivatized with 100 μL of BSTFA reagent (1% TMCS, v/v) at 37°C for 30 min. The derivatized solution was diluted with n‐hexane to a final volume of 1 mL and filtered through a 0.22 μm organic‐phase syringe filter. GC–MS analysis was conducted using an Agilent 8890–5977B (Agilent Technologies, Wilmington, Delaware, USA) gas chromatograph. A 1 μL aliquot of the sample was injected in the splitless mode. Helium was used as the carrier gas at a flow rate of 1.2 mL/min. The initial temperature was maintained at 40°C for 1 min, then increased to 100°C at a rate of 20°C/min, then increased to 300°C at 15°C/min and held for 5 min. The temperatures of transfer line and ion source were set to 280 and 230°C, respectively. In the electron impact mode, the energy was 70 eV.

### 2.9. Statistical Analysis

The data were presented as the mean ± SEM. The normality of data distribution was assessed using the Shapiro–Wilk test to ensure compliance with the assumptions underlying parametric statistical tests. The *p*‐values for all variables were greater than 0.05, indicating that the normality assumption was satisfied. Homogeneity of variance was evaluated using Levene’s test, and all variables also showed *p*  > 0.05, indicating homogeneous variances among groups. All data were subjected to one‐way analysis of variance (ANOVA) using SPSS 25.0 (SPSS Inc., Chicago, IL, USA). Within the ANOVA framework, polynomial contrasts, including linear and quadratic trends, were applied to examine dose‐dependent responses. When significant effects were detected, Tukey’s multiple range test was used for post hoc multiple comparisons, with *p*  < 0.05 defined as the level of statistical significance. Regression assumptions were checked during polynomial regression analysis, and the rationale for regression modeling was justified based on the biological response patterns observed. Graphical representations were performed using GraphPad Prism 8.0 (GraphPad Software, San Diego, CA, USA).

## 3. Results

### 3.1. Whole Fish Growth Performance and Nutrient Composition

The growth performance and morphological indices of LMB are presented in Table 2. The substitution of different proportions of FM by BFLM exerted no significant influence on SR (*p* > 0.05). The FBW, WGR, SGR, FER, PER, VSI, and HSI were significantly affected both linearly and quadratically by dietary BFLM levels (*p* < 0.05). Furthermore, also no differences were founded in FBW, WGR, SGR, and FER between the control group and the BFLM17 group (*p* > 0.05), and then decreased with the increasing BFLM replacement levels to 68% (*p* < 0.05). The HSI and VSI presented an increasing trend from BFLM17 to BFLM68 group (*p* < 0.05).

**Table 2:** The growth performance, morphological indices, and whole fish nutrient compositions of LMB (*n* = 3).

Items	Diet groups					SEM	ANOVA	Line		Quadratic	
	BFLM0	BFLM17	BFLM34	BFLM51	BFLM68		*p*‐Value	*p*‐Value	Adj.*R* ^2^	*p*‐Value	Adj.*R* ^2^
IBW (g)	25.99	25.98	25.98	25.97	25.97	0.01	0.90	0.54	−0.03	0.83	−0.09
FBW (g)	106.50^a^	108.01^a^	89.60^b^	88.93^b^	89.45^b^	2.21	0.02	0.00	0.59	0.00	0.61
WGR (%)	296.72^ab^	302.26^a^	244.87^b^	242.40^b^	238.70^b^	0.69	0.03	0.00	0.65	0.00	0.66
SGR (%/d)	2.46^a^	2.49^a^	2.21^b^	2.20^b^	2.18^b^	0.03	0.03	0.00	0.64	0.00	0.64
SR (%)	96.88	96.88	100.00	100.00	98.44	0.62	0.28	0.16	0.06	0.18	0.09
FER	1.05^a^	1.07^a^	0.87^b^	0.86^b^	0.84^b^	0.24	0.00	0.00	0.69	0.00	0.69
VSI (%)	6.73^a^	5.91^b^	5.89^b^	5.88^b^	6.33^ab^	0.11	0.03	0.32	0.00	0.01	0.26
HSI (%)	1.25^a^	0.98^b^	1.00^b^	0.99^b^	1.05^ab^	0.03	0.01	0.06	0.09	0.01	0.28
RGL (%)	80.75	80.07	80.84	80.68	80.61	0.83	1.00	0.97	−0.03	0.99	−0.06
CF (g/cm^3^)	2.41	2.36	2.41	2.36	2.40	0.01	0.74	0.90	−0.14	0.84	−0.02
Moisture (%)	71.45	71.52	72.29	72.10	71.53	0.16	0.32	0.48	−0.03	0.03	0.33
Crude fat (%)	7.59^a^	7.30^a^	6.59^b^	6.52^b^	6.75^b^	0.11	0.00	0.00	0.38	0.00	0.61
Crude protein (%)	16.50	16.87	16.65	16.42	16.45	0.09	0.56	0.40	−0.01	0.49	−0.03

*Note*: Values in the same column with different superscript letters are significantly different (*p* < 0.05).

Abbreviations: CF, condition factor; FBW, final body weight; FER, feed efficiency rate; HSI, hepatosomatic indices; IBW, initial body weight; RGL, relative gut length; SGR, specific growth rate; SR, survival rate; VSI, viscerosomatic index; WGR, weight gain rate.

For whole fish nutrient composition, data were presented in Table 2. Crude fat content was linearly and quadratically affected by BFLM level (*p* < 0.05), but there was no difference between the control and the BFLM17 group (*p* > 0.05), and decreased with increasing replacement levels up to 51% (*p* < 0.05). No significant difference was found in moisture and crude protein content in fish diet different feeds (*p* > 0.05).

### 3.2. Liver Health

#### 3.2.1. Plasma Physiology and Biochemistry

The effects of BFLM replacing FM on plasma physiological and biochemical indicators are presented in Table 3. Plasma TG, HDL, and LDL were significantly affected both linearly and quadratically by BFLM replacement level (*p* < 0.05). TP, CHOL, CLU, and HDL increased significantly with increasing BFLM replacement up to 17% (*p* < 0.05), and then decreased thereafter. TG content was significantly increased in the BFLM51 group compared to other groups (*p* < 0.05). LDL levels increased continuously with rising BFLM replacement levels (*p* < 0.05).

**Table 3:** The effects of BFLM replacing fishmeal on plasma physiological and biochemical indicators (*n* = 3).

Items	Diet groups					SEM	ANOVA	Line		Quadratic	
	BFLM0	BFLM17	BFLM 34	BFLM51	BFLM68		*p*‐Value	*p*‐Value	Adj.*R* ^2^	*p*‐Value	Adj.*R* ^2^
TP (g/L)	25.65^c^	30.08^a^	26.15^c^	27.85^b^	26.35^bc^	1.71	0.00	0.77	−0.05	0.22	0.07
CHOL (mmol/L)	4.86^b^	5.40^a^	4.63^c^	4.63^c^	4.81^b^	0.38	0.00	0.15	0.07	0.20	0.08
TG (mmol/L)	2.16^b^	2.49^ab^	2.65^ab^	2.71^a^	2.54^ab^	0.23	0.00	0.00	0.35	0.02	0.32
CLU (mmol/L)	28.45^c^	31.57^a^	31.36^a^	30.94^ab^	29.54^bc^	2.61	0.00	0.72	−0.05	0.00	0.71
HDL (mmol/L)	3.10^b^	3.40^a^	2.73^d^	2.87^c^	2.85^c^	0.25	0.00	0.01	0.32	0.02	0.29
LDL (mmol/L)	0.86^d^	1.05^bc^	1.09^b^	1.01^c^	1.17^a^	0.12	0.00	0.00	0.49	0.00	0.52

*Note:* Values in the same column with different superscript letters are significantly different (*p* < 0.05).

#### 3.2.2. Liver Histology and TG Concentration

**Figure 1:**
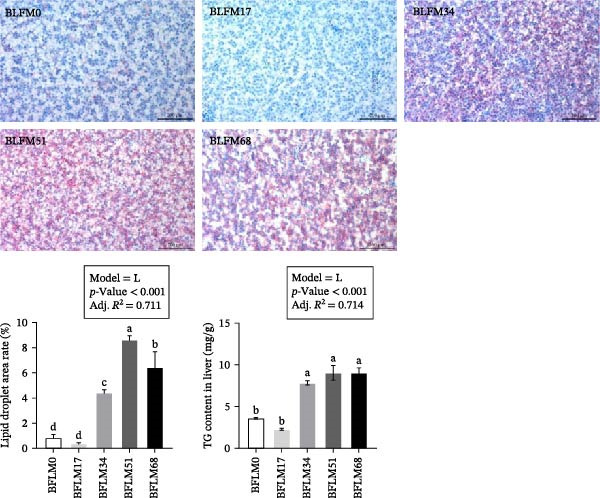
Oil red O stained liver sections and TG content in liver (*n* = 3). Values with different lowercase letters are significantly different (*p* < 0.05).

Oil red O staining and hepatic TG concentration exhibited patterns consistent with hepatic lipid accumulation (Figure 1). No significant difference was discovered between the control and BFLM17 group (*p* > 0.05). However, the number of red lipid droplets in liver sections and TG concentration were highest for the BFLM51 group and the BFLM68 group (*p* < 0.05), respectively, and both parameters were linearly affected by dietary BFLM level (*p* < 0.05).

#### 3.2.3. Glucolipid Metabolism

**Figure 2:**
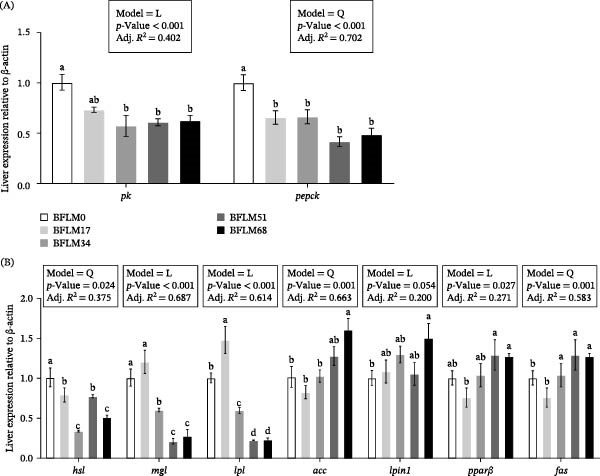
The gene expression levels of key enzymes and factors related to glucolipid metabolism in liver (*n* = 6). (A) Relative mRNA expression of glycolysis‐related genes (*pk* and *pepck*), (B) relative mRNA expression of lipid metabolism‐related genes (*hsl*, *mgl*, *lpl*, *acc*, *lpin1*, *pparβ* and *fas*). BFLM0, 17, 34, 51, 68 represent 0%, 17%, 34%, 51%, and 68% black soldier fly larval meal (BFLM) replacement levels, respectively. The measured genes include: *pk* (pyruvate kinase), *pepck* (phosphoenolpyruvate carboxykinase), *hsl* (hormone sensitive lipase), *mgl* (monoglyceride lipase), *lpl* (lipoprotein lipase), *acc* (acetyl‐CoA carboxylase), *lpin1* (lipin 1), *pparβ* (peroxisome proliferator‐activated receptor β), and *fas* (fatty acid synthase). Values with different lowercase letters are significantly different at *p* < 0.05. Model = *L*, Linear regression model; Model = *Q*, Quadratic regression model. The y‐axis shows the gene expression level relative to β‐actin.

The expression levels of key enzymes and factors related to glucolipid metabolism are shown in Figure 2. The expression of *pk*, *mgl*, *lpl*, and *pparβ* was linearly affected by dietary BFLM level (*p* < 0.05), whereas *pepck*, *hsl*, *acc*, and *fas* were quadratically affected (*p* < 0.05). Compared with the control group, *pk* mRNA levels had no significant difference in the BFLM17 group, but was reduced in the other substitution groups (*p* < 0.05). Likewise, pepck expression was significantly decreased in all BFLM substitution groups compared with the control group (*p* < 0.05). For FAs metabolism, *mgl*, *acc*, *lpin1*, *pparβ*, and *fas* expression levels in the BFLM17 group showed no significant difference with BFLM0 group (*p* > 0.05). High BFLM replacement levels (BFLM51 and BFLM68) activated the gene expression of the key enzymes in lipid anabolism (*pparβ* and *fas*), while inhibited the gene expression of the key enzymes in lipid catabolism (*hsl*, *mgl*, and *lpl*) (*p* < 0.05).

#### 3.2.4. Antioxidant and Immune Performance

##### 3.2.4.1. ANS Biosynthesis

**Figure 3:**
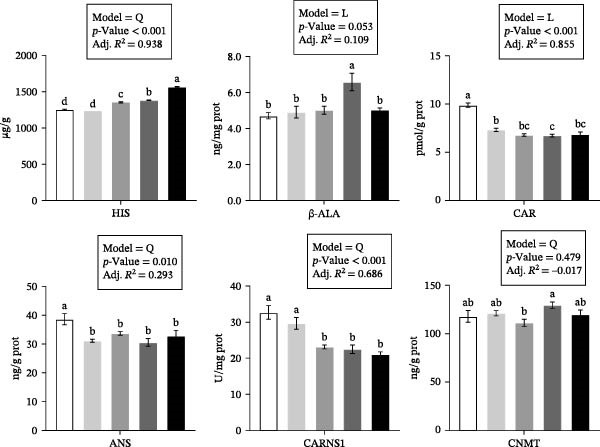
Changes of enzymes and productions of CAR and ANS synthesis pathways in muscle (*n* = 3). Values with different lowercase letters are significantly different (*p* < 0.05).

As shown in Figure 3, the concentrations of His, ANS and CARNS1 were quadratically affected by dietary BFLM level (*p* < 0.05), whereas CAR was linearly affected (*p* < 0.05). The concentration of His in muscle increased with increasing BFLM replacement level up to 68% (*p* < 0.05), although no significant difference was observed between the control and BFLM17 group (*p* > 0.05). The concentration of β‐ALA in muscle reached its highest value when 51% of FM replaced by BFLM (*p* < 0.05). Conversely, the concentration of CAR and ANS decreased progressively with increasing BFLM replacement level (*p* < 0.05). Moreover, the activities of CARNS1 decreased when FM replacement by BFLM exceeded 34% (*p* < 0.05).

##### 3.2.4.2. Antioxidant Status

**Figure 4:**
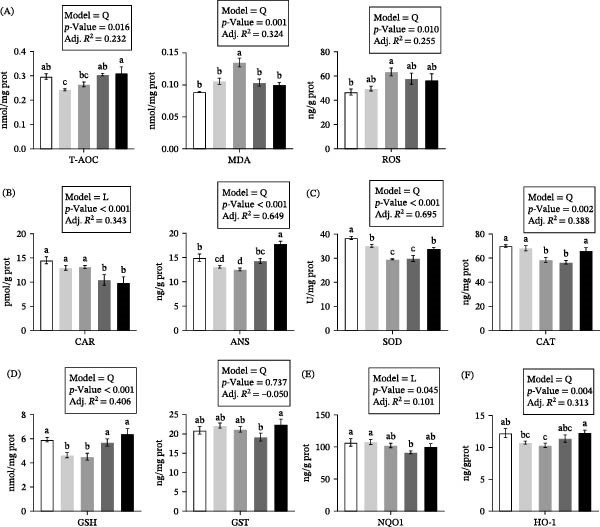
The key enzymes and active substances involved in the antioxidant status of the liver, including (A) total antioxidant capacity (T‐AOC), malondialdehyde (MDA) and reactive oxygen species (ROS), (B) carnosine (CAR) and anserine (ANS), (C) superoxide dismutase (SOD), catalase (CAT), (D) glutathione (GSH), glutathione S‐transferase (GST), (E) NAD(P)H quinone dehydrogenase 1 (NQO1), and (F) heme oxygenase‐1 (HO‐1) (*n* = 3). Values with different lowercase letters are significantly different (*p* < 0.05).

Figure 4 shows the key enzymes and active substances involved in antioxidant status of liver. T‐AOC, MDA, ROS, ANS, SOD, CAT, GSH, and HO‐1 were quadratically affected by BFLM replacement levels (*p* < 0.05), whereas CAR and NQO1 were linearly affected (*p* < 0.05). ROS and MDA showed an increase‐then‐decrease pattern, with the highest level observed in the BFLM34 group. T‐AOC decreased as the BFLM replacement level increased to 17% and then increased thereafter. Moreover, the content of CAR in liver was significantly lower in 51% and 68% replacement levels (*p* < 0.05). The concentration of ANS, SOD, CAT, GSH, GST, NQO1, and HO‐1 decreased to the lowest at 34% or 51% replacement, then increased (*p* < 0.05).

##### 3.2.4.3. nrf2‐keap1 Signaling Pathway

**Figure 5:**
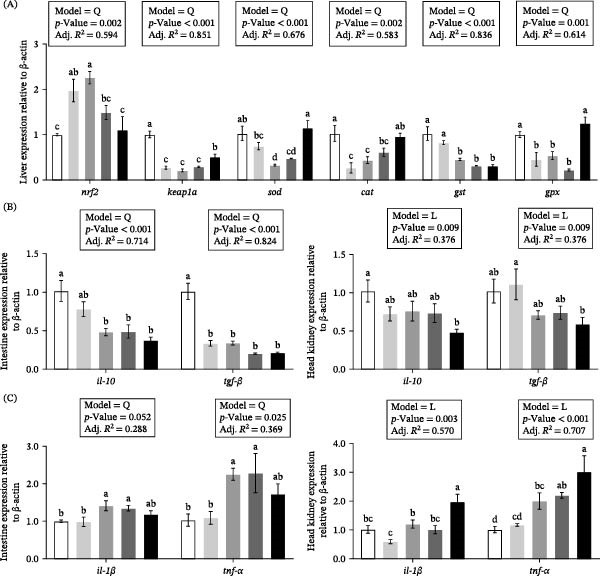
The liver’s mRNA expression levels of the key regulators involved in nrf2‐keap1 signaling pathway (A), and the effect of BFLM replacements on mRNA expression level of anti‐inflammatory (B), and pro‐inflammatory (C) factors in intestine and head kidney (*n* = 6). The measured genes include: *nrf2* (nuclear factor erythroid 2‐related factor 2), *keap1* (kelch‐like ECH‐associated protein 1), *sod* (superoxide dismutase), *cat* (catalase), *gst* (glutathione S‐transferase), *gpx* (glutathione peroxidase), *il-10* (interleukin‐10), *tgf-β* (transforming growth factor‐β), *il-1β* (interleukin‐1β), and *tnf-α* (tumor necrosis factor‐α). Values with different lowercase letters are significantly different at *p* < 0.05.

The liver’s mRNA expression levels of the key regulators involved in the Nrf2‐Keap1 signaling pathway are presented in Figure 5. The expression levels of *nrf2*, *keap1a*, *sod*, *cat*, *gst*, and *gpx* were quadratically affected by dietary BFLM level (*p* < 0.05). *Nrf2* and *keap1a* showed contrary expression patterns. Compared with the control group, the BFLM34 group showed significantly higher *nrf2* expression and lower *keap1a* expression (*p* < 0.05). The gene expression levels of *sod*, *cat*, *gst*, and *gpx* in liver had the similar pattern, BFLM34, BFLM17, BFLM51, and BFLM68 had the lower mRNA expression levels of *sod*, *cat*, *gst*, and *gpx*, respectively (*p* < 0.05).

##### 3.2.4.4. Immune Response

**Figure 6:**
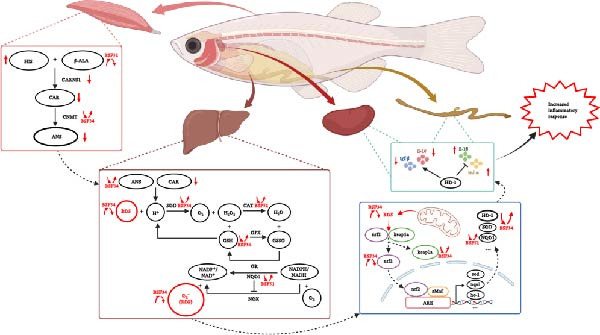
Partial antioxidant and immune performance after FM replaced by BFLM on LMB.

The effects of dietary BFLM replacement on the mRNA expression levels of anti‐inflammatory and pro‐inflammatory key factors in the intestine and head kidney are presented in Figure 5. *Il-10*, *tgf-β*, and *tnf-α* in intestine were quadratically affected by BFLM substitution (*p* < 0.05); *il-10*, *tgf-β*, *il-1β*, and *tnf-α* in head kidney were linearly affected (*p* < 0.05). *Il-10* and *tgf-β* mRNA levels in different tissues showed a similar pattern. BFLM substitution decreased their expression levels in the intestine and head kidney (*p* < 0.05). Compared with the control group, the BFLM17 group had no significant difference on the *il-1β* and *tnf-α* (*p* > 0.05), whereas higher substitutions significantly increased the expression of these pro‐inflammatory factors in all tissues (*p* < 0.05). The overall antioxidant and immune performance of LMB following the replacement of FM with BFLM is summarized in Figure 6.

### 3.3. Muscle Growth and Quality

#### 3.3.1. Muscle Nutritional Composition

The nutrient component of muscle is shown in Table 4. Muscle crude fat, crude protein and H‐Pro contents were significantly affected both linearly and quadratically by dietary BFLM level (*p* < 0.05). Muscle moisture content was not significantly affected by BFLM substitution (*p* > 0.05). Likewise, compared with the control diet, the BFLM17 diet also did not significantly affect the contents of crude fat, crude protein, and H‐Pro in muscle (*p* > 0.05). However, exceeding BFLM17, crude fat and crude protein contents showed a decreasing trend with increasing BFLM replacement levels. The lowest content of crude fat and crude protein were observed in the BFLM51 group, while the lowest H‐Pro content was observed in the BFLM34 and 68 groups (*p* < 0.05).

**Table 4:** Nutrient components of muscle (%, *n* = 3).

Items	Diet groups					SEM	ANOVA	Line		Quadratic	
	BFLM0	BFLM17	BFLM 34	BFLM51	BFLM68		*p*‐Value	*p*‐Value	Adj.*R* ^2^	*p*‐Value	Adj.*R* ^2^
Moisture	77.77	78.16	77.74	77.59	77.47	0.54	0.47	0.18	0.05	0.31	0.03
Crude fat	1.19^a^	1.13^a^	1.10^ab^	0.88^b^	0.96^ab^	0.15	0.00	0.00	0.42	0.01	0.40
Crude protein	19.58^a^	19.54^a^	19.23^ab^	19.06^b^	19.28^ab^	0.29	0.03	0.01	0.27	0..02	0.32
H‐Pro	5.61^a^	4.97^a^	3.59^b^	4.07^b^	3.79^b^	0.23	0.00	0.00	0.53	0.00	0.64

*Note:* Values in the same column with different superscript letters are significantly different (*p* < 0.05).

#### 3.3.2. Muscle AAs Concentration and Transportation

**Figure 7:**
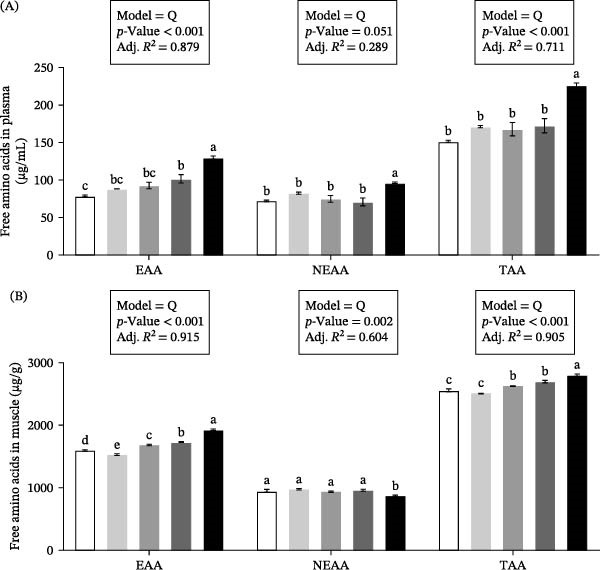
Free amino acid (FAA) concentrations in (A) plasma and (B) muscle, including essential amino acids (EAA), non‐essential amino acids (NEAA), and total amino acids (TAA) (*n* = 3). Values with different lowercase letters are significantly different (*p* < 0.05).

The FAA concentrations in plasma and muscle are shown in Figure 7. The levels of essential AA (EAA), nonessential AA (NEAA), and total AA (TAA) in muscle, as well as EAA and TAA in plasma, were quadratically affected by BFLM level (*p* < 0.05). In plasma, EAA increased progressively with increasing BFLM replacement level up to 68%, whereas NEAA and TAA were significantly elevated only in the BFLM68 group (*p* < 0.05). Similarly, in muscle, BFLM substitutions elevated the concentrations of EAA and TAA, which reached their highest levels in fish fed the BFLM 68 diet (*p* < 0.05) but declined to their lowest levels in fish fed the BFLM17 diet (*p* < 0.05). However, NEAA concentration decreased with increasing BFLM replacement levels up to 68% (*p* < 0.05).

**Figure 8:**
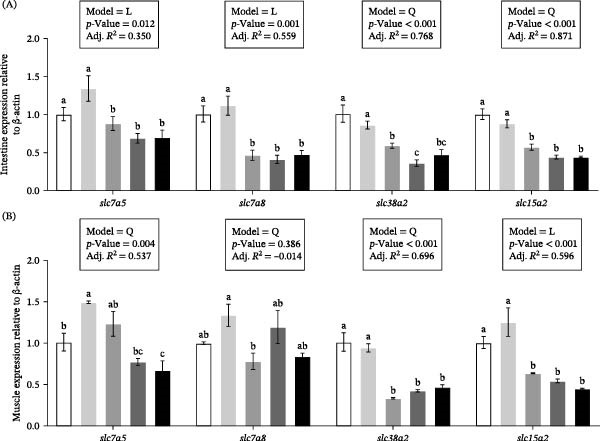
mRNA expression level of AAs and small peptide transporters in (A) intestine and (B) muscle (*n* = 6). The measured transporters include: *slc7a5* (solute carrier family 7 member 5), *slc7a8* (solute carrier family 7 member 8), *slc38a2* (solute carrier family 38 member 2), and *slc15a2* (solute carrier family 15 member 2). Values with different lowercase letters are significantly different at *p* < 0.05.

Additionally, the mRNA expression levels of genes related to AAs and small peptide transport in the intestine and muscle are presented in Figure 8. *Slc7a5* and *slc7a8* in intestine and *slc15a2* in muscle were linearly affected by BFLM substitution (*p* < 0.05), *slc38a2* and *slc15a2* in intestine and *slc7a5* and *slc38a2* in muscle were quadratically affected (*p* < 0.05). In the intestine, the mRNA expression levels of *slc7a5*, *slc7a8*, *slc38a2*, and *slc15a2* did not differ significantly between the BFLM17 and control groups (*p* > 0.05). However, when the substitution rate exceeded 17%, these transporter genes were significantly downregulated (*p* < 0.05). A similar pattern was observed in muscle, 17% replacement did not inhibited the gene expression level of these transporters (*p* > 0.05), whereas the BFLM51 and BFLM68 groups exhibited markedly lower expression levels of *slc7a5*, *slc38a2*, and *slc15a2* than the control group (*p* < 0.05).

#### 3.3.3. Muscle Nutrient Metabolism

**Figure 9:**
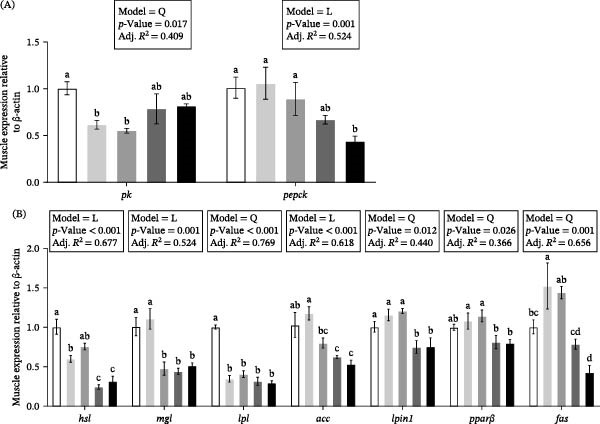
The gene expression level of key regulatory factors and enzymes related to glucolipid metabolism in muscle (*n* = 6). (A) mRNA expression of glycolysis‐related genes in muscle. (B) mRNA expression of lipid metabolism‐related genes in muscle. The measured genes include: *pk* (pyruvate kinase), *pepck* (phosphoenolpyruvate carboxykinase), *hsl* (hormone‐sensitive lipase), *mgl* (monoglyceride lipase), *lpl* (lipoprotein lipase), *acc* (acetyl‐CoA carboxylase), *lpin1* (lipin 1), *pparβ* (peroxisome proliferator‐activated receptor β), and *fas* (fatty acid synthase). Values with different lowercase letters are significantly different at *p* < 0.05.

The expression levels of key regulatory factors and enzymes related to glycolysis and fatty metabolism in muscle are shown in Figure 9. The expression levels of *pk*, *lpl*, *lpin1*, *pparβ*, and *fas* were quadratically affected by different BFLM level (*p* < 0.05), whereas *pepck*, *hsl*, *mgl*, and *acc* were linearly affected (*p* < 0.05). *Pk* expression decreased continuously as the BFLM replacement increased to 34% (*p* < 0.05) and then increased thereafter. Moreover, *pepck* expression level was reduced in the higher substitution groups (BFLM51 and BFLM68) (*p* < 0.05). Lipid catabolism‐related key factors were significantly lower in BFLM34, BFLM51, and BFLM68 groups than in the BFLM0 group (*p* < 0.05), whereas no significant difference in *mgl* expression was observed between the control and BFLM17 groups (*p* > 0.05). Genes related to lipid anabolism also presented a decreasing trend following BFLM substitution (*p* < 0.05). Specifically, the BFLM51 and BFLM68 diets significantly restricted the expression of *acc*, *lpin1*, and *ppar-β*, with the lowest levels observed in fish fed the BFLM68 diet (*p* < 0.05). However, BFLM17 showed no significant difference compared with the control diet (*p* > 0.05).

#### 3.3.4. Muscle Nutrient Sensing Signaling Pathway Regulation

**Figure 10:**
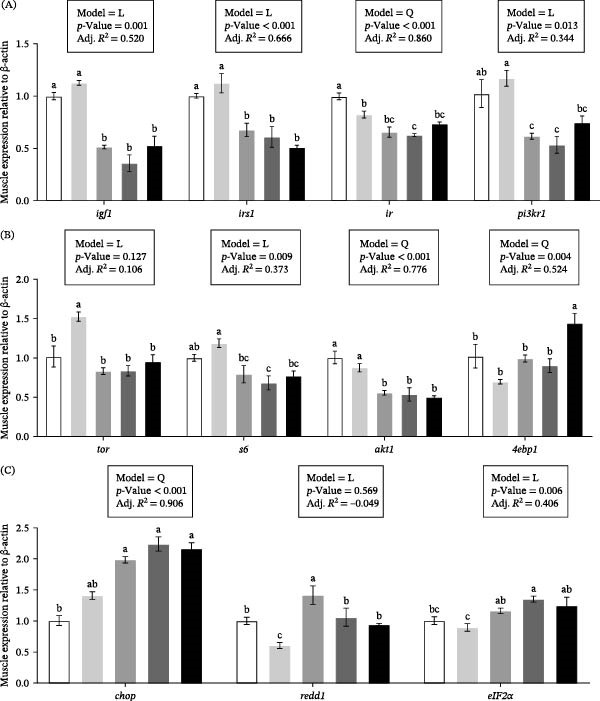
The gene expression level of key factors related to the (A) GH‐IGF, (B) TOR, and (C) AAR signaling pathways (*n* = 6). The measured genes include: *igf1* (insulin‐like growth factor 1), *ir* (insulin receptor), *irs1* (insulin receptor substrate 1), *pi3kr1* (phosphatidylinositol 3‐kinase regulatory subunit 1), *akt1* (protein kinase B 1), *tor* (target of rapamycin), *s6* (ribosomal protein S6), *redd1* (regulated in development and DNA damage responses 1), *4ebp1* (eukaryotic translation initiation factor 4E‐binding protein 1), *eIF2α* (eukaryotic initiation factor 2α), and *chop* (C/EBP homologous protein). Values with different lowercase letters are significantly different at *p* < 0.05.

The mRNA expression levels of key factors involved in the GH‐IGF, TOR, and AAR signaling pathways are shown in Figure 10. The expression levels of *igf1*, *irs1*, *pi3kr1*, *tor*, *s6*, *redd1*, and *eIF2α* were linearly affected by dietary BFLM level (*p* < 0.05), whereas *ir*, *akt1*, *4ebp1*, and *chop* were quadratically affected (*p* < 0.05). No significant differences were observed in the expression levels of *igf1*, *irs1*, *pi3kr1*, *s6*, *akt1*, *4ebp1*, and *eIF2α* between the control group and BFLM17 group (*p* > 0.05). However, higher BFLM replacement levels inhibited the GH‐IGF and TOR signaling pathways while activating the AAR signaling pathway. Lower expression levels of *igf1*, *irs1*, *Ir*, *pi3kr1*, and *akt1*, together with higher expression levels of *chop and eIF2α*, were observed when FM replacement by BFLM exceeded 34% or 51% (*p* < 0.05).

#### 3.3.5. Muscle Texture Profiles

Muscle texture indicators including hardness, adhesiveness, cohesiveness, springiness, gumminess, and chewiness were concerned in the present study (Table 5). Hardness, adhesiveness, gumminess, and chewiness were significantly affected both linearly and quadratically by dietary BFLM level (*p* < 0.05). BFLM substitution decreased hardness, gumminess, and chewiness in a dose‐dependent manner, with the lowest values observed in the BFLM68 group. However, adhesion of muscle showed the opposite trend. No significant differences were found in springiness, gumminess, and chewiness among the control, BFLM17 and BFLM34 groups (*p* > 0.05).

**Table 5:** Indicators of muscle texture (*n* = 6).

Items	Diet groups					SEM	ANOVA	Line		Quadratic	
	BFLM0	BFLM17	BFLM 34	BFLM51	BFLM68		*p*‐Value	*p*‐Value	Adj.*R* ^2^	*p*‐Value	Adj.*R* ^2^
Hardness (N)	21.76^ab^	25.50^a^	17.61^bc^	17.98^bc^	14.10^c^	4.88	0.00	0.00	0.39	0.00	0.40
Adhesion (N·mm)	0.05^d^	0.07^cd^	0.15^a^	0.13^ab^	0.10^bc^	0.04	0.00	0.02	0.23	0.00	0.68
Cohesiveness	0.21^ab^	0.18^b^	0.26^a^	0.17^b^	0.21^ab^	0.05	0.03	0.69	−0.04	0.87	−0.09
Springiness (mm)	2.16	2.12	2.21	1.87	1.81	0.29	0.17	0.05	0.13	0.11	0.11
Gumminess (N)	4.49^a^	4.60^a^	4.54^a^	3.03^b^	2.92^b^	1.07	0.01	0.00	0.37	0.00	0.38
Chewiness (mJ)	9.72^a^	9.95^a^	9.90^a^	5.68^b^	5.40^b^	2.93	0.01	0.00	0.37	0.00	0.39

*Note:* Values in the same column with different superscript letters are significantly different (*p* < 0.05)

#### 3.3.6. Muscle Fiber Growth and Differentiation

**Figure 11:**
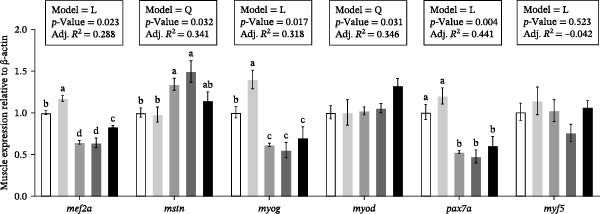
mRNA expression level of key factors in the growth and differentiation of muscle fiber (*n* = 6). The measured genes include: *mef2a* (myocyte enhancer factor 2A), *myog* (myogenin), *myod* (myogenic differentiation 1), *myf5* (myogenic factor 5), *pax7a* (paired box 7a), and *mstn* (myostatin). Values with different lowercase letters are significantly different at *p* < 0.05.

The mRNA expression levels of key factors in the growth and differentiation of muscle fiber are shown in Figure 11. *Mef2a*, *myog*, and *pax7a* were linearly affected by BFLM level (*p* < 0.05), whereas *mstn and myod* were quadratically affected (*p* < 0.05). *Mef2a* and *myog* expression levels were highest in fish fed the BFLM replacing 17% of FM compared with the control group (*p* < 0.05). No significant differences were found in *myod*, *pax7a*, *myf5*, and *mstn* expression levels between the BFLM0 and BFLM17 groups (*p* > 0.05). However, 34%, 51%, and 68% BFLM replacements levels downregulated the gene expression levels of *mef2a*, *myog*, and *pax7a* (*p* < 0.05). *Mstn* expression level presented an increasing trend with 34%–68% substitutions (*p* < 0.05).

#### 3.3.7. Muscle Volatile Metabolites Change Pattern

**Figure 12:**
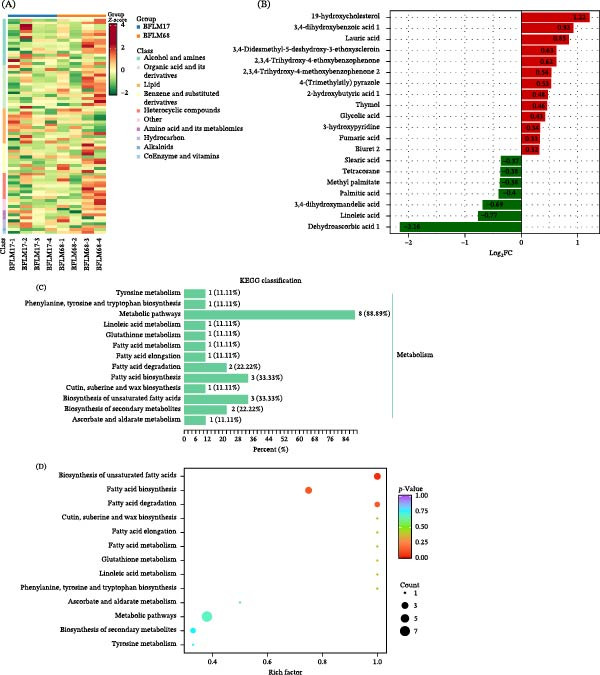
Effects of fishmeal replacement by BFLM on volatile metabolites. (A) Heatmap of differential volatile metabolites. (B) Volcano plot of differential volatile metabolites. (C) KEGG pathway classification enrichment bar chart. (D) Bubble plot of enriched metabolic pathways. The figure shows the key differential metabolites and related metabolic pathways, including tyrosine, linoleic acid, phenylalanine, glutathione (GSH), fatty acid (FA) metabolism, ascorbate and aldarate metabolism, and secondary metabolite biosynthesis. A total of 20 differential metabolites were identified between the two groups, with 13 upregulated and 7 downregulated in the BFLM68 group compared to the BFLM17 group (*p* < 0.05).

Based on growth performance results, the effects of FM replacement by BFLM on muscle volatile metabolites were further investigated through comparative analyses between the BFLM17 (minimal impact) and BFLM68 groups (maximal impact) (Figure 12). KEGG analysis showed that, compared with the BFLM17 group, the BFLM68 group exhibited significant differences in the metabolic pathways, including tyrosine, linoleic acid, phenylalanine, GSH, FA metabolism, and secondary metabolite biosynthesis including ascorbate and aldarate metabolism (*p* < 0.05). A total of 20 differential metabolites were identified between the two groups. Compared with BFLM17, BFLM68 significantly elevated 13 metabolites and reduced seven metabolites. These volatile metabolites predominantly enriched in the biosynthesis of unsaturated FAs, FA synthesis and degradation pathways (*p* < 0.05). Further analysis of the 13 upregulated metabolites revealed that they were primarily alcohols and ketones derived from FA oxidation and amines produced from AA degradation. Meanwhile, seven downregulated metabolites mainly included volatile flavor aromatic compounds, such as FAs.

#### 3.3.8. Flavor Compounds of Muscle

The bound AA and FA composition of muscle are shown in Table 6. Except for Met and His, all bound AAs in muscle were linearly and quadratically affected (*p* < 0.05). For UAA, compared with the control diet, the BFLM17 diet did not significantly altered the contents of Asp, Glu, and ∑UAA (*p* > 0.05). However, all of them showed a decreasing trend with increasing BFLM replacement levels, and the lowest content was presented in BFLM68 group (*p* < 0.05). Moreover, similar trends were also found in Thr, Ser, ∑SAA, Val, Tyr, Arg, and ∑AA contents. Compared with fish fed the control diet, fish fed the BFLM17 diet showed no significant difference in Thr, Ser, ∑SAA, Val, Tyr, Arg, or ∑AA contents (*p* > 0.05). However, fish fed the BFLM34, BFLM51, and BFLM68 showed significantly lower content of these flavor‐contributing AA (*p* < 0.05).

**Table 6:** Bound amino acid compositions in the muscle (%, *n* = 6).

Items	Diet groups					SEM	ANOVA	Line		Quadratic	
	BFLM0	BFLM17	BFLM 34	BFLM51	BFLM68		*p*‐Value	*p*‐Value	Adj.*R* ^2^	*p*‐Value	Adj.*R* ^2^
Asp	6.42^a^	5.94^a^	4.70^b^	4.70^b^	4.62^b^	0.92	0.01	0.00	0.57	0.00	0.62
Glu	9.58^a^	8.86^ab^	7.02^bc^	7.64^bc^	6.70^c^	0.36	0.02	0.00	0.48	0.01	0.48
∑UAA	16.00^a^	14.82^ab^	11.72^c^	12.24^bc^	11.40^c^	0.59	0.01	0.00	0.53	0.00	0.55
Thr	2.86^a^	2.60^ab^	2.10^b^	2.42^ab^	2.04^b^	0.11	0.04	0.01	0.38	0.03	0.36
Ser	2.48^a^	2.22^ab^	1.80^bc^	1.96^bc^	1.74^c^	0.09	0.02	0.00	0.48	0.01	0.51
Gly	2.50	2.52	2.20	1.90	2.00	0.09	0.07	0.01	0.42	0.02	0.37
Ala	2.44	2.22	1.82	2.08	1.80	0.09	0.09	0.02	0.32	0.04	0.31
∑SAA	15.92^a^	14.52^ab^	11.49^c^	12.42^bc^	11.38^c^	0.58	0.01	0.02	0.50	0.00	0.54
Val	2.32^a^	2.18^a^	1.78^b^	1.76^b^	1.68^b^	0.08	0.01	0.00	0.57	0.00	0.59
Met	1.66	1.92	1.44	1.20	1.14	0.13	0.30	0.05	0.22	0.14	0.16
Ile	2.22^a^	2.24^a^	1.70^b^	1.68^b^	1.86^ab^	0.08	0.03	0.02	0.31	0.02	0.38
Leu	5.00^a^	4.62^a^	3.76^b^	3.64^b^	3.78^b^	0.14	0.02	0.01	0.40	0.02	0.39
Tyr	3.40^a^	2.96^ab^	2.38^b^	2.96^ab^	2.30^b^	0.03	0.01	0.01	0.35	0.04	0.33
Lys	6.42	6.16	5.20	5.44	4.96	0.20	0.08	0.01	0.41	0.02	0.38
His	2.36	2.56	2.34	2.22	2.18	0.07	0.48	0.15	0.09	0.30	0.05
Arg	5.62^a^	5.44^a^	4.60^ab^	4.80^ab^	4.22^b^	0.19	0.04	0.01	0.43	0.02	0.39
∑BAA	32.52^a^	30.82^a^	23.42^b^	27.42^ab^	24.44^b^	1.17	0.02	0.01	0.36	0.03	0.37
∑AA	64.44^a^	60.16^ab^	46.63^c^	52.21^bc^	47.23^c^	2.30	0.02	0.00	0.45	0.01	0.47

*Note*: AA, total binding amino acids. Values in the same column with different superscript letters are significantly different (*p* < 0.05).

Abbreviations: BAA, bitter amino acids; SAA, sweet amino acids; UAA, umami amino acids.

**Figure 13:**
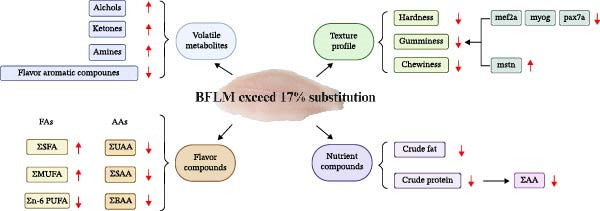
The overall changes of muscle quality after BLFM exceed 17% substitutions.

Furthermore, six SFAs, five MUFAs, and six PUFAs were detected in the present study (Table 7). Except for C17:0, C18:0, C24:0, C15:1, C22:6n‐3, and Σn‐3 PUFA, all FAs in muscle were linearly and quadratically affected (*p* < 0.05). The proportions of C12:0, C14:0, C16:0, and ΣSFA in muscle increased with increasing BFLM replacement level up to 68% (*p* < 0.05). Similar trends were observed for C16:1, C20:1, ΣMUFA, C20:3n‐6, C20:4n‐6, and Σn‐3 PUFA/Σn‐6 PUFA ratio (*p* < 0.05). Conversely, the proportions of C18:3n–3, C18:2n–6c, and Σn‐6 PUFA decreased with increasing BFLM substitution level. Unexpectedly, the levels of these indicators in the BFLM17 group showed no significant differences compared with those in the control group. The overall changes of muscle quality when BFLM substitution exceeded 17% are summarized in Figure 13.

**Table 7:** Composition of fatty acids in the muscle (%, *n* = 6).

Items	Diet groups					SEM	ANOVA	Line		Quadratic	
	BFLM0	BFLM17	BFLM 34	BFLM51	BFLM68		*p*‐Value	*p*‐Value	Adj.*R* ^2^	*p*‐Value	Adj.*R* ^2^
C12:0	0.39^c^	0.94^c^	1.42^bc^	2.29^ab^	2.78^a^	0.82	0.00	0.00	0.86	0.00	0.85
C14:0	1.11^d^	1.50^cd^	1.92^bc^	2.44^ab^	2.70^a^	0.55	0.00	0.00	0.87	0.00	0.86
C16:0	20.04^c^	21.10^b^	22.02^ab^	22.32^a^	22.96^a^	0.97	0.00	0.00	0.87	0.00	0.89
C17:0	0.29	0.30	0.30	0.28	0.28	0.11	0.39	0.54	−0.05	0.00	0.62
C18:0	6.48	6.50	6.46	6.19	6.47	0.28	0.20	0.74	−0.07	0.09	0.23
C24:0	0.62	0.61	0.58	0.53	0.58	0.05	0.33	0.40	−0.02	0.37	0.01
ΣSFA	28.99^d^	31.13^c^	32.91^b^	33.63^b^	35.19^a^	2.23	0.00	0.00	0.92	0.00	0.91
C16:1	1.66^c^	1.83^bc^	2.32^ab^	2.68^a^	2.80^a^	0.51	0.00	0.00	0.87	0.00	0.86
C15:1	0.43	0.45	0.53	0.51	0.55	0.09	0.78	0.76	−0.07	0.40	0.00
C18:1n9t	0.42^b^	0.51^ab^	0.80^a^	0.60^ab^	0.68^ab^	0.14	0.00	0.23	0.04	0.01	0.48
C18:1n9c	24.60	24.71	25.89	27.15	27.25	1.42	0.01	0.00	0.64	0.00	0.61
C20:1	0.92^c^	0.99^c^	1.24^b^	1.29^ab^	1.36^a^	0.20	0.00	0.00	0.93	0.00	0.94
MUFA	27.92^b^	28.43^b^	31.28^a^	31.34^a^	32.29^a^	2.06	0.00	0.00	0.83	0.00	0.82
C18:3n‐3	2.11^a^	1.87^b^	1.28^c^	1.24^c^	1.00^d^	0.41	0.00	0.00	0.89	0.00	0.92
DHA (C22:6n‐3)	5.40	5.60	5.66	5.52	5.76	0.59	0.82	0.06	0.19	0.07	0.24
Σn‐3 PUFA	7.62	7.72	7.16	7.29	7.22	0.56	0.17	0.57	−0.05	0.19	0.11
C18:2n‐6c	30.87^a^	28.72^a^	24.49^b^	22.08^c^	19.59^d^	4.50	0.00	0.00	0.97	0.00	0.97
C18:3n‐6	0.38^b^	0.32^b^	0.49^a^	0.37^b^	0.34^b^	0.06	0.00	0.44	−0.03	0.27	0.06
C20:3n‐6	0.55^c^	0.57^bc^	0.82^abc^	0.80^ab^	0.85^a^	0.17	0.00	0.00	0.77	0.00	0.76
C20:4n‐6	1.99^b^	2.66^ab^	2.27^ab^	2.46^ab^	3.12^a^	0.54	0.01	0.03	0.25	0.10	0.21
Σn‐6 PUFA	33.2^a^	32.13^a^	28.86^b^	26.34^c^	23.10^d^	4.09	0.03	0.00	0.95	0.00	0.95
Σn‐3 PUFA/Σn‐6 PUFA	0.23^b^	0.23^b^	0.24^a^	0.28^a^	0.29^a^	0.04	0.00	0.00	0.69	0.00	0.79

*Note:* Values in the same column with different superscript letters are significantly different (*p* < 0.05).

Abbreviations: MUFA, monounsaturated fatty acid; PUFA, polyunsaturated fatty acid; SFA, saturated fatty acid.

## 4. Discussion

The present study investigated the effects of replacing FM with BFLM in the diet of *Micropterus salmoides* from three perspectives: growth performance, liver health, and muscle growth and quality. Based on the analysis of SGR and FER, the recommended substitution level of BFLM for FM in the diet was no more than 17%. This result was consistent with previous studies on turbot (*Scophthalmus maximus*) and grass carp (*Ctenopharyngodon idellus*), which reported impaired growth performance following high‐level replacement of FM with BFLM [25, 26]. Further analysis of fish morphological indices revealed that higher substitution of FM with BFLM significantly altered VSI and HSI, with the changes in HSI being the most pronounced. This suggests that high BFLM substitution may exert adverse effects on liver health [27]. Moreover, data on whole‐body nutrient compositions showed a significant decrease in the crude fat content following substitution, implying a disruption of lipid metabolism. It is well known that liver is the primary organ for lipid metabolism in vertebrates [28], and subsequent analyses focused on alterations in liver homeostasis in response to BFLM replacement of dietary FM.

The physiological and biochemical indicators in plasma serve as direct indicators of tissues and organs homeostasis status. Accordingly, these parameters were first measured to assess hepatic health homeostasis. It was found that FM replacement with BFLM significantly increased the concentration of TP, CHOL, TG, GLU, HLD, and LDL in plasma. These results further support previous findings that high dietary inclusion levels of BFLM induce marked alterations in nutrient metabolism, particularly lipid metabolism [29]. More intuitively, histological examination of the liver revealed that the number of lipid droplets in the BFLM34, BFLM51, and BFLM68 groups were significantly higher than those in the control group. Consistent with the histological findings, the concentration of TG in liver was also significantly increased in these groups. These results indicate that under high levels of BFLM replacement, lipids were not effectively transported and utilized but instead accumulated in the liver, ultimately linking to fatty liver in LMB.

By analyzing the reasons, we found that the FA composition of BFLM revealed that it contains over 50% SFA. In contrast, the content of LCFA was only 10%, and the n‐3/n‐6 ratio is only 0.15. This profile differs markedly from that of FM, which contained more than 30% LCFA and had an n‐3/n‐6 ratio greater than 16% (Tables S1–S3). Further analysis of the expression level of key enzymes and regulators involved in glucolipid metabolism showed that high levels of BFLM replacement promoted lipid synthesis while suppressing lipid catabolism and glycolysis, ultimately contributing to increased hepatic lipid deposition [30]. Collectively, these data suggest that the higher the BFLM inclusion level, the greater the deficiency in effectively utilizable lipids. To compensate, the organism appeared to enhance lipid synthesis capacity while reducing lipid utilization, thereby aggravating hepatic lipid accumulation. Therefore, replacing FM with BFLM at levels exceeding 17% may cause a marked imbalance in dietary FA composition, fail to meet physiological requirements, and ultimately be predictive of excessive lipid accumulation in the liver and disruption of metabolic homeostasis.

As the body’s major metabolic and biosynthetic organ, the liver plays essential roles not only in nutrient metabolism but also in antioxidant defense, detoxification, and immune regulation [31]. Hence, the present study also focused on changes in the antioxidant–immune system after diet BFLM substitution. Surprisingly, during the analysis of FAA in plasma and muscle, His showed one of the most pronounced changes. In addition to its role in protein synthesis, His is also a precursor for CAR and ANS, two bioactive peptides that contribute importantly to ROS scavenging [32]. In general, ROS homeostasis is maintained by both enzymatic and nonenzymatic antioxidant systems. SOD, GST, and GPx are key components of the enzymatic antioxidant system, whereas CAR and ANS are important active molecules in the nonenzymatic system [33, 34]. They complement each other and are both indispensable for maintaining redox homeostasis.

Data in the present study showed that all BFLM substitution groups had lower CAR and ANS levels than the control group, whereas ROS and MDA levels in the liver were higher in the BFLM34 group. These results suggest that the reduction in CAR and ANS associated with BFLM substitution disrupted the antioxidant balance and was linked to elevated ROS levels in the liver. Further examination of the antioxidant defense system indicated that when the organism sensed increased oxidative stress, the enzymatic antioxidant system was activated in a compensatory manner. Therefore, in the high‐substitution groups (BFLM51 and BFLM68), we observed higher activities of antioxidant‐related factors such as SOD, GST, and GSH than in the other groups. Similar findings have been reported in piglets and poultry, further supporting the important roles of CAR and ANS in the antioxidant–immune system [35, 36].

In addition, the key factors involved in the Nrf2‐Keap1 and immune response pathways were also analyzed. Higher levels of BFLM substitution appeared to induce oxidative stress, which in turn activated the Nrf2‐Keap1 pathway as a compensatory antioxidant response, accompanied by the upregulation of antioxidant‐regulated genes such as *sod* and *cat*. However, this adaptive response may have been insufficient to fully counteract the elevated oxidative stress, as indicated by the concomitant reduction in anti‐inflammatory factors, such as *tgf-β* and *il-10*, and the increased expression of pro‐inflammatory factors such as *il-1β* and *tnf-α*. It is important to note that activation of *nrf2* itself does not directly cause inflammation; rather, the inflammatory response observed here was more likely a consequence of persistent oxidative stress and possibly the activation of parallel inflammatory pathways such as NF‐κB signaling [37]. The exacerbated inflammatory response ultimately impaired the stress resistance of the fish. Consistent with the conclusions based on growth performance, our integrated antioxidant–immune data further suggest that the substitution level of BFLM for FM should not exceed 17%. This threshold may represent a critical balance point at which the compensatory antioxidant response remains sufficient to control oxidative stress without provoking excessive inflammatory injury.

In addition to stress resistance, muscle deposition and quality are among the most important economic traits used to evaluate the effectiveness of novel protein sources as replacements for FM [38]. In the present study, muscle nutrient composition data showed that BFLM substitution levels exceeding 17% significantly reduced crude protein and H‐Pro contents, indicating detrimental effects on muscle growth and quality. Therefore, this aspect was also systematically investigated.

The concentrations of FAA in plasma and muscle are closely associated with protein turnover [39]. In the present study, BFLM substitution significantly increased the levels of FAA in both plasma and muscle. However, further analysis of the expression levels of AA transporters showed that the relevant transporters were downregulated in the substitution groups. These results suggest that FAAs in the body could not be effectively transported, utilized, or deposited [20]. As discussed earlier, higher levels of BFLM substitution also triggered inflammatory responses, which may place the body in a relatively high metabolic state and accelerate protein breakdown, thereby further increasing FAA levels [40]. Taken together, these observations support the view that high levels of BFLM substitution adversely affect protein metabolism. In addition to protein metabolism, the present study also found that substitution levels exceeding 17% suppressed anabolic processes and altered lipid and GLU metabolism through regulation of nutrient‐sensing (GH‐IGF, TOR, and AAR) signaling pathways. These changes likely reduced the deposition of major nutrients in muscle and ultimately contributed to lower body weight [41]. Therefore, the negative effects of high BFLM replacement on muscle growth appear to arise from a combined reduction in the deposition of the three major nutrients: protein, carbohydrate, and lipid.

In recent years, increasing attention has been given to the muscle quality of farmed fish. The evaluation of muscle quality is a complex and systematic issue [42]. Texture characteristics, volatile compounds, flavor substances, and nutritional composition are all important indicators of quality [43]. Since the present study evaluated all of these aspects, the effects of BFLM substitution on muscle quality could be comprehensively characterized. With respect to texture, the data showed that the BFLM17 diet had no significant effects on muscle texture compared with the control diet, whereas substitution levels exceeding 17% markedly reduced hardness, gumminess, and chewiness while increasing adhesiveness. Further analysis of muscle fiber growth‐related genes showed that 34%–68% BFLM substitutions reduced the expression of positive regulators associated with muscle growth (*mef2a*) and differentiation (*myog*, *myod*, *pax7a*, and *myf5*) while increasing the expression of negative regulator *mstn*. This linked to an increase in the number of muscle fibers but inhibited muscle fiber growth resulting in a decrease in muscle hardness, gumminess, and chewiness.

On the other hand, the present study conducted metabolomics analysis to investigate changes in volatile compounds in muscle [44]. Compared with the BFLM17 group, the BFLM68 group showed more pronounced alterations in the lipid metabolism. Further analysis revealed that the BFLM68 group had significantly higher levels of alcohols, ketones, and amines but markedly lower levels of various FAs derived volatile aromatic compounds. Alcohols, ketones, and amines are mainly generated through the oxidative degradation of FAs and AAs in muscle and are generally regarded as off‐flavor compounds [45]. Therefore, these findings indicate that high levels of BFLM substitution reduced desirable volatile flavor substances while increasing off‐flavor compounds in muscle, thereby negatively affecting muscle quality.

Bound AAs also contribute substantially to muscle flavor and quality. Different AAs produce distinct taste attributes; for example, Asp and Glu contribute umami, whereas Thr, Ser, and Gly contribute sweetness [46]. Our analysis showed that replacing FM with BFLM significantly altered the profile of bound AAs in muscle, which are important contributors to flavor. When the substitution level exceeded 34%, the contents of UAAs and SAAs decreased significantly, indicating that a high BFLM substitution reduced umami‐ and sweet‐tasting compounds in the muscle and may therefore diminish palatability. This finding is consistent with studies in Pacific white shrimp (*Litopenaeus vannamei*) and triploid crucian carp, in which high replacement of FM by alternative proteins also adversely affected flavor‐related compounds [47, 48]. In addition to bound AAs, FA composition is another key determinant of muscle flavor and consumer acceptance [49]. In the present study, BFLM substitution increased the contents of SFA and MUFA while reducing n‐6 PUFA. This shift likely resulted from the high SFA content of BFLM itself, which may have been deposited into muscle under high dietary inclusion levels. Excessive SFA accumulation in muscle may negatively affect quality in at least two ways. First, it may increase the risk of undesirable flavor formation, as high SFA content can contribute to the production of undesirable volatile compounds during processing or heating. Second, it may impair collagen synthesis and thereby adversely affect muscle texture. Therefore, determining an appropriate substitution level for BFLM is crucial. Based on our comprehensive evaluation of muscle quality indicators, we conclude that the replacement of FM with BFLM in LMB diets should not exceed 17%.

## 5. Conclusion

**Figure 14:**
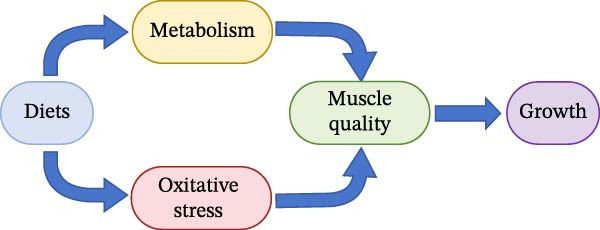
The integrated effects of dietary manipulation on metabolism, oxidative stress, muscle quality, and growth performance.

In summary, our findings demonstrate that, based on a systematic evaluation, the optimal substitution level of BFLM in diets for LMB should not exceed 17%. First, replacement levels above 17% reduced SGR, FER, and whole‐body crude lipid content, thereby impairing the growth performance. Second, these higher substitution levels disrupted the antioxidant system, induced oxidative stress, and promoted inflammatory responses, ultimately compromising stress resistance. Furthermore, BFLM replacement above 17% reduced muscle hardness and chewiness as well as the contents of aroma‐, umami‐, and sweet‐related compounds while increasing the levels of off‐flavor substances and SFAs, thereby making the muscle more susceptible to oxidative deterioration. Comprehensive analysis indicated that the imbalance in FA composition was a significant factor limiting the ability of BFLM to replace FM at high inclusion levels. To improve the efficiency of BFLM as an alternative to FM, its FA composition should be optimized, while growth performance, antioxidant‐immune status, and muscle quality should all be considered comprehensively. The integrated biological cascade of these effects is illustrated in Figure 14, which summarizes how dietary BFLM substitution modulates metabolism and oxidative stress status, thereby influencing muscle quality and ultimately determining growth performance in LMB.

## Author Contributions

Yang Chen was responsible for conducting the feeding trial. Hang Su, Xiangyu She, and Ruyi Li were responsible for analyzing the experimental samples. Xirui Li was responsible for writing – original draft, formal analysis, and data analysis. Kangsen Mai was responsible for designing experiment and revising the manuscript. Xinyu Li was responsible for designing experiment. Fei Song was responsible for revising the manuscript, supervision, and funding acquisition.

## Funding

This work obtained financial support from the National Key Research and Development Program of China (Grants 2023YFD2400600 and 2024YFD2402000), the National Natural Science Foundation of China (Grant 32403037), the Guangdong Province Natural Science Foundation Project (Grants 2024A1515013001 and 2026A1515010380), the Key Laboratory of Mariculture, Ministry of Education (Ocean University of China), and the Specialized Innovation Project of Ordinary Universities in Guangdong Province (Grant 2021KQNCX017).

## Disclosure

All authors have read and agreed to the published version of the manuscript. A preprint has previously been published [50].

## Ethics Statement

The procedures used in this study strictly complied with the regulations of the University Animal Care and Use Committee of South China Normal University (Approval Reference Number SCNU‐SLS‐2025‐060).

## Conflicts of Interest

Xinyu Li is an employee of the Guangdong Yuehai Feed Group Co., Ltd. The other authors declare no conflicts of interest.

## Supporting Information

Additional supporting information can be found online in the Supporting Information section.

## Supporting information


**Supporting Information** The Supporting Information presents the FA compositions of FM and BFLM. Table S1: The FAs compositions of the ingredients used in the feeds. Table S2: The AAs compositions of the ingredients used in the feeds. Table S3: Primer sequences used for real‐time quantitative PCR.

## Data Availability

The data that support the findings of this study are available from the corresponding author upon reasonable request.

## References

[1] Troell M. , Costa-Pierce B. , and Stead S. , et al.Perspectives on Aquaculture’s Contribution to the Sustainable Development Goals for Improved Human and Planetary Health, Journal of the World Aquaculture Society. (2023) 54, no. 2, 251–342, 10.1111/jwas.12946.

[2] Cooney R. , Wan A. H. L. , O’Donncha F. , and Clifford E. , Designing Environmentally Efficient Aquafeeds Through the Use of Multicriteria Decision Support Tools, Current Opinion in Environmental Science & Health. (2021) 23, 10.1016/j.coesh.2021.100276, 100276.

[3] Hua K. , Cobcrof J. M. , and Cole A. , et al.The Future of Aquatic Protein: Implications for Protein Sources in Aquaculture Diets, One Earth. (2019) 1, no. 3, 316–329, 10.1016/j.oneear.2019.10.018.

[4] Hunter M. C. , Smith R. G. , Schipanski M. E. , Atwood L. W. , and Mortensen D. A. , Agriculture in 2050: Recalibrating Targets for Sustainable Intensification, Bioscience. (2017) 67, no. 4, 386–391, 10.1093/biosci/bix010.

[5] Freccia A. , Sergio Bee Tubin J. , Nishioka Rombenso A. , and Gustavo Coelho Emerenciano M. , Insects in Aquaculture Nutrition: An Emerging Eco-Friendly Approach or Commercial Reality?, 2020, Intechopen, 1–14.

[6] Guerreiro I. , Serra C. R. , and Coutinho F. , et al.Digestive Enzyme Activity and Nutrient Digestibility in Meagre (*Argyrosomus regius*) Fed Increasing Levels of Black Soldier Fly Meal (*Hermetiaillucens*), Aquaculture Nutrition. (2021) 27, no. 1, 142–152, 10.1111/anu.13172.

[7] Kim S. W. , Less J. F. , and Wang L. , et al.Meeting Global Feed Protein Demand: Challenge, Opportunity, and Strategy, Annual Review of Animal Biosciences. (2019) 7, no. 1, 221–243, 10.1146/annurev-animal-030117-014838. 30418803

[8] de Verdal H. , Komen H. , and Quillet E. , et al.Improving Feed Efficiency in Fish Using Selective Breeding: A Review, Reviews in Aquaculture. (2018) 10, no. 4, 833–851, 10.1111/raq.12202.

[9] Mohan K. , Rajan D. K. , and Muralisankar T. , et al.Use of Black Soldier Fly (*Hermetiaillucens* L.) Larvae Meal in Aquafeeds for a Sustainable Aquaculture Industry: A Review of Past and Future Needs, Aquaculture. (2022) 553, 10.1016/j.aquaculture.2022.738095, 738095.

[10] Kim T.-K. , Yong H. I. , Kim Y.-B. , Kim H.-W. , and Choi Y.-S. , Edible Insects as a Protein Source: A Review of Public Perception, Processing Technology, and Research Trends, Food Science of Animal Resources. (2019) 39, no. 4, 521–540, 10.5851/kosfa.2019.e53. 31508584 PMC6728817

[11] Kawasaki K. , Hashimoto Y. , and Hori A. , et al.Evaluation of Black Soldier Fly (*Hermetiaillucens*) Larvae and Pre-Pupae Raised on Household Organic Waste, as Potential Ingredients for Poultry Feed, Animals. (2019) 9, no. 3, 98–111, 10.3390/ani9030098. 30893879 PMC6466380

[12] Li M. M. , Li M. F. , and Wang G. Y. , et al.Defatted Black Soldier Fly (*Hermetia illucens*) Larvae Meal can Partially Replace Fish Meal in Diets for Adult Chinese Soft-Shelled Turtles, Aquaculture. (2021) 541, 10.1016/j.aquaculture.2021.736758, 736758.

[13] Gonçalves L. U. , de Oliveira J. B. , and de Dantas F. M. , et al.Defatted Black Soldier Fly Meal in Diets for Juvenile Pirarucu, *Arapaima gigas*: Digestibility, Growth Performance and Health Parameters, Aquaculture. (2025) 598, 10.1016/j.aquaculture.2024.742071, 742071.

[14] Zhang T. H. , Zhang L. Z. , and Yin T. , et al.Recent Understanding of Stress Response on Muscle Quality of Fish: From the Perspective of Industrial Chain, Trends in Food Science & Technology. (2023) 140, 10.1016/j.tifs.2023.104145, 104145.

[15] Wang W. Q. , Yang P. , and He C. Q. , et al.Effects of Dietary Methionine on Growth Performance and Metabolism Through Modulating Nutrient-Related Pathways in Largemouth Bass (*Micropterus salmoides*), Aquaculture Reports. (2021) 20, 10.1016/j.aqrep.2021.100642, 100642.

[16] Yang P. , Wang W. Q. , and Chi S. Y. , et al.Effects of Dietary Lysine on Regulating GH-IGF System, Intermediate Metabolism and Immune Response in Largemouth Bass (*Micropterus salmoides*), Aquaculture Reports. (2020) 17, 10.1016/j.aqrep.2020.100323, 100323.

[17] Fischer H. , Romano N. , Renukdas N. , Kumar V. , and Sinha A. K. , Comparing Black Soldier Fly (*Hermetiaillucens*) Larvae Versus Prepupae in the Diets of Largemouth Bass, *Micropterus salmoides*: Effects on Their Growth, Biochemical Composition, Histopathology, and Gene Expression, Aquaculture. (2022) 546, 10.1016/j.aquaculture.2021.737323, 737323.

[18] Xu F. M. , Hou S. W. , and Wang G. X. , et al.Effects of Zymolytic Black Soldier Fly (*Hermetiaillucens*) Pulp as Dietary Supplementation in Largemouth Bass (*Micropterus salmoides*), Aquaculture Reports. (2021) 21, 10.1016/j.aqrep.2021.100823, 100823.

[19] Wang P. , Yan X. F. , and Zhang X. T. , et al.Increasing Levels of Fishmeal Replacement by Defatted Black Soldier Fly Larvae Meal Reduced Growth Performance Without Affecting Fillet Quality in Largemouth Bass (*Micropterus salmoides*), Fish Physiology and Biochemistry. (2024) 50, no. 6, 2255–2274, 10.1007/s10695-024-01390-x. 39083156

[20] Wang W. , Xu Y. , Chi S. , Yang P. , Mai K. , and Song F. , Dietary Lysine Regulates Body Growth Performance via the Nutrient-Sensing Signaling Pathways in Largemouth Bass (*Micropterus salmoides*), Frontiers in Marine Science. (2020) 7, 10.3389/fmars.2020.595682, 595682.

[21] Xu H. , Ai Q. , and Mai K. , et al.Effects of Dietary Arachidonic Acid on Growth Performance, Survival, Immune Response and Tissue Fatty Acid Composition of Juvenile Japanese Seabass, *Lateolabrax japonicus* , Aquaculture. (2010) 307, no. 1-2, 75–82, 10.1016/j.aquaculture.2010.07.001.

[22] Crouse J. D. , Calkins C. R. , and Seideman S. C. , The Effects of Rate of Change in Body Weight on Tissue Development and Meat Quality of Youthful Bulls, Journal of Animal Science. (1986) 63, no. 6, 1824–1829, 10.2527/jas1986.6361824x. 3818463

[23] Geng H. Y. , Yang P. , and Chen Y. , et al.Dietary Choline Can Partially Spare Methionine to Improve the Feeds Utilization and Immune Response in Juvenile Largemouth Bass (*Micropterus salmoides*): Based on Phenotypic Response to Gene Expression, Aquaculture Reports. (2023) 30, 10.1016/j.aqrep.2023.101546, 101546.

[24] Zhang B. , Cao M. L. , and Wang X. D. , et al.The Combined Analysis of GC-IMS and GC-MS Reveals the Differences in Volatile Flavor Compounds Between Yak and Cattle-Yak Meat, Foods. (2024) 13, no. 15, 10.3390/foods13152364, 2364. 39123555 PMC11311445

[25] Zhao J. , Pan J. , Zhang Z. , Chen Z. , Mai K. , and Zhang Y. , Fishmeal Protein Replacement by Defatted and Full-Fat Black Soldier Fly Larvae Meal in Juvenile Turbot Diet: Effects on the Growth Performance and Intestinal Microbiota, Aquaculture Nutrition. (2023) 2023, 10.1155/2023/8128141, 8128141. 37089257 PMC10115534

[26] Hu Z. , Li H. , Liu S. , Xue R. , Sun J. , and Ji H. , Assessment of Black Soldier Fly (*Hermetiaillucens*) Larvae Meal as a Potential Substitute for Soybean Meal on Growth Performance and Flesh Quality of Grass Carp *Ctenopharyngodon idellus* , Animal Nutrition. (2023) 14, 425–449, 10.1016/j.aninu.2023.06.006. 37649678 PMC10463206

[27] Kari Z. A. , Téllez-Isaías G. , and Hamid N. K. A. , et al.Effect of Fish Meal Substitution With Black Soldier Fly (*Hermetiaillucens*) on Growth Performance, Feed Stability, Blood Biochemistry, and Liver and Gut Morphology of Siamese Fighting Fish (*Betta splendens*), Aquaculture Nutrition. (2023) 2023, 10.1155/2023/6676953, 6676953. 39553242 PMC11401699

[28] Ponziani F. R. , Pecere S. , Gasbarrini A. , and Ojetti V. , Physiology and Pathophysiology of Liver Lipid Metabolism, Expert Review of Gastroenterology & Hepatology. (2015) 9, no. 8, 1055–1067, 10.1586/17474124.2015.1056156. 26070860

[29] Chen Y. K. , Chi S. Y. , and Zhang S. , et al.Effect of Black Soldier Fly (*Hermetiaillucens*) Larvae Meal on Lipid and Glucose Metabolism of Pacific White Shrimp *Litopenaeusvannamei* , British Journal of Nutrition. (2022) 128, no. 9, 1674–1688, 10.1017/S0007114521004670. 34814963

[30] Hu Y. J. , Huang Y. H. , and Tang T. , et al.Effect of Partial Black Soldier Fly (*Hermetia illucens* L.) Larvae Meal Replacement of Fish Meal in Practical Diets on the Growth, Digestive Enzyme and Related Gene Expression for Rice Field Eel (*Monopterus albus*), Aquaculture Reports. (2020) 17, 10.1016/j.aqrep.2020.100345, 100345.

[31] Li S. , Tan H. Y. , and Wang N. , et al.The Role of Oxidative Stress and Antioxidants in Liver Diseases, International Journal of Molecular Sciences. (2015) 16, no. 11, 26087–26124, 10.3390/ijms161125942. 26540040 PMC4661801

[32] Brosnan M. E. and Brosnan J. T. , Histidine Metabolism and Function, The Journal of Nutrition. (2020) 150, 2570S–2575S, 10.1093/jn/nxaa079. 33000155 PMC7527268

[33] Ighodaro O. M. and Akinloye O. A. , First Line Defence Antioxidants-Superoxide Dismutase (SOD), Catalase (CAT) and Glutathione Peroxidase (GPX): Their Fundamental Role in the Entire Antioxidant Defence Grid, Alexandria Journal of Medicine. (2018) 54, no. 4, 287–293, 10.1016/j.ajme.2017.09.001.

[34] Boldyrev A. A. , Aldini G. , and Derave W. , Physiology and Pathophysiology of Carnosine, Physiological Reviews. (2013) 93, no. 4, 1803–1845, 10.1152/physrev.00039.2012. 24137022

[35] Wu G. Y. , Important Roles of Dietary Taurine, Creatine, Carnosine, Anserine and 4-Hydroxyproline in Human Nutrition and Health, Amino Acids. (2020) 52, no. 3, 329–360, 10.1007/s00726-020-02823-6. 32072297 PMC7088015

[36] Derave W. , De Courten B. , and Baba S. P. , An Update on Carnosine and Anserine Research, Amino Acids. (2019) 51, no. 1, 1–4, 10.1007/s00726-018-02689-9. 30617755

[37] Reis-Mendes A. , Ferreira M. , and Padrão A. I. , et al.The Role of Nrf2 and Inflammation on the Dissimilar Cardiotoxicity of Doxorubicin in Two-Time Points: A Cardio-Oncology In Vivo Study Through Time, Inflammation. (2024) 47, no. 1, 264–284, 10.1007/s10753-023-01908-0. 37833616 PMC10799157

[38] Zhou Z. , Yao W. , Ye B. , Wu X. , Li X. , and Dong Y. , Effects of Replacing Fishmeal Protein With Poultry By-Product Meal Protein and Soybean Meal Protein on Growth, Feed Intake, Feed Utilization, Gut and Liver Histology of Hybrid Grouper (*Epinephelus fuscoguttatus*♀ × *Epinephelus lanceolatus*♂) Juveniles, Aquaculture. (2020) 516, 10.1016/j.aquaculture.2019.734503, 734503.

[39] Li Y. H. , Wei H. K. , and Li F. N. , et al.Regulation in Free Amino Acid Profile and Protein Synthesis Pathway of Growing Pig Skeletal Muscles by Low-Protein Diets for Different Time Periods, Journal of Animal Science. (2016) 94, no. 12, 5192–5205, 10.2527/jas.2016-0917. 28046182

[40] Klasing K. C. and Austic R. E. , Changes in Protein Degradation in Chickens Due to an Inflammatory Challenge, Experimental Biology and Medicine. (1984) 176, no. 3, 292–296, 10.3181/00379727-176-41873. 6374671

[41] Caputo M. , Pigni S. , and Agosti E. , et al.Regulation of GH and GH Signaling by Nutrients, Cells. (2021) 10, no. 6, 10.3390/cells10061376, 1376. 34199514 PMC8227158

[42] Zhang Z. Y. , Limbu S. M. , and Zhao S. H. , et al.Dietary l-Carnitine Supplementation Recovers the Increased pH and Hardness in Fillets Caused by High-Fat Diet in Nile Tilapia (*Oreochromis niloticus*), Food Chemistry. (2022) 382, 10.1016/j.foodchem.2022.132367, 132367. 35152027

[43] Zhang Z. Y. , Jiang Z. Y. , and Lv H. B. , et al.Dietary Aflatoxin Impairs Flesh Quality Through Reducing Nutritional Value and Changing Myofiber Characteristics in Yellow Catfish (*Pelteobagrus fulvidraco*), Animal Feed Science and Technology. (2021) 274, 10.1016/j.anifeedsci.2020.114764, 114764.

[44] Li J. , Tang C. H. , and Zhao Q. Y. , et al.Integrated Lipidomics and Targeted Metabolomics Analyses Reveal Changes in Flavor Precursors in Psoas Major Muscle of Castrated Lambs, Food Chemistry. (2020) 333, 10.1016/j.foodchem.2020.127451, 127451. 32683255

[45] McGorrin R. , Character Impact Compounds: Flavors and Off-Flavors in Food, Flavor, Fragrance, and Odor Analysis, 2001, CRC Press, 391–430.

[46] Brand J. G. , Teeter J. H. , Kumazawa T. , Huque T. , and Bayley D. L. , Transduction Mechanisms for the Taste of Amino Acids, Physiology & Behavior. (1991) 49, no. 5, 899–904, 10.1016/0031-9384(91)90201-X. 1679559

[47] Cai L. , Bai J. , Lan Y. , Song F. , and Wei Z. , Effects of Composite Mixture of Protein Sources in Replacing Fish Meal on Nutritional Value and Flavor Quality of Pacific White Shrimp (*Litopenaeus vannamei*), Aquaculture Reports. (2023) 28, 10.1016/j.aqrep.2022.101437, 101437.

[48] Yang L. Q. , Yi C. L. , and Mo Y. J. , et al.Effects of Different Protein Sources on Growth Performance, Muscle Flavor Substances and Quality Structure in Triploid Crucian Carp, Fishes. (2024) 9, no. 1, 23–36, 10.3390/fishes9010023.

[49] Wood J. D. , Richardson R. I. , and Nute G. R. , et al.Effects of Fatty Acids on Meat Quality: A Review, Meat Science. (2004) 66, no. 1, 21–32, 10.1016/S0309-1740(03)00022-6. 22063928

[50] Chen Y. , Li X. , and Li X. , et al.Effects of Black Soldier Fly (*hermetia illucens* L.) Larvae Meal Replaced Fishmeal in Largemouth Bass (*micropterus salmoides*): A Comprehensive Analysis of Growth, Antioxidant-Immune Response, and Muscle Quality, 2025, Social Science Research Network 10.2139/ssrn.5616554.

