# Supplementary material for: A Comprehensive Analysis of Dietary Fishmeal Replaced by Black Soldier Fly (Hermetia illucens L.) in Largemouth Bass (Micropterus salmoides)
Source: Aquac Nutr. 2026 Jun 25;2026:7583815. doi: 10.1155/anu/7583815 (PMC13296239; doi:10.1155/anu/7583815)
Supplement: Supplementary file 1 — Supporting Information The Supporting Information presents the FA compositions of FM and BFLM. Table S1: The FAs compositions of the ingredients used in the feeds. Table S2: The AAs compositions of the ingredients used in the feeds. Table S3: Primer sequences used for real‐time quantitative PCR. [file ANU-2026-7583815-s001.docx]

Supplementary table 1: The FAs compositions of the ingredients used in the feeds

| FAs | BLFM | FM |
| --- | --- | --- |
| C10:0 | 0.00 | 0.00 |
| C12:0 | 28.60 | 0.10 |
| C14:0 | 9.51 | 5.83 |
| C16:0 | 12.35 | 20.90 |
| C18:0 | 2.01 | 0.33 |
| **ΣSFA** | **52.47** | **27.17** |
| C16:1 | 3.76 | 5.15 |
| C18:1 | 25.21 | 8.25 |
| **ΣMUFA** | **28.97** | **13.40** |
| C18:2 n-6 | 12.50 | 1.19 |
| C18:3 n-6 | 0.00 | 0.76 |
| C18:3 n-3 | 1.54 | 0.18 |
| C20:4 n-6 | 0.10 | 1.09 |
| C20:5 | 0.30 | 11.10 |
| DHA | 0.00 | 20.30 |
| **ΣPUFA** | **14.44** | **34.62** |
| **n-3/n-6** | **0.15** | **16.23** |
| Total | 95.88 | 75.18 |

Supplementary table 2: The AAs compositions of the ingredients used in the feeds

| AAs | BLFM | FM |
| --- | --- | --- |
| Asp | 3.52 | 6.05 |
| Thr | 1.45 | 2.82 |
| Ser | 1.46 | 2.54 |
| Glu | 4.05 | 8.45 |
| Gly | 1.81 | 3.95 |
| Ala | 2.47 | 4.13 |
| Val | 2.04 | 3.23 |
| Met | 0.72 | 1.94 |
| Ile | 1.52 | 2.69 |
| Leu | 2.50 | 4.75 |
| Tyr | 2.62 | 2.02 |
| Phe | 1.51 | 2.76 |
| Lys | 2.54 | 5.32 |
| His | 1.13 | 2.13 |
| Arg | 1.91 | 3.86 |
| Pro | 1.83 | 2.65 |
| Total | 33.08 | 59.29 |

| Supplementary table 3: Primer sequences used for real-time quantitative PCR | | |
| --- | --- | --- |
| **Genes** | **Forward primer (5’-3’)** | **Reverse primer (5’-3’)** |
|  |  |  |
| ***Glucolipid metabolism signaling pathway*** | | |
| *pk* | CTCTTTCATCCGCAAAGC | AATTCCCAGGTCACCACG |
| *pepck* | GGAAACGGCCAACATTCT | GCCAACCAGCAGTTCTCAT |
| *hsl* | ATCAGAGCTGGAGCACCCTA | GCAGAGGAGAGCAGAAAGGA |
| *mgl* | AAGGTTTTTCTGGCGAAGGT | CGTGGAAGTTCAGCTCATCA |
| *lpl* | TTCCTCGACCCTCTGAAAGA | GGAGTCAAGTTTGCCAGGAA |
| *acc* | ATCCCTCTTTGCCACTGTTG | GAGGTGATGTTGCTCGCATA |
| *lpin1* | TCCTACGTTCCCGAGAGAAA | TACGAGGGAACCACTTCCTG |
| *pparβ* | AGCACCTCGCCATTTGTAATCT | GGACCCCAATCTCCTTCGTC |
| *fas* | CACCGTAGAACCGAGCCCGCT | CGCCATGAAGATCCTAAAGAA |
| ***Antioxidant -Immune system*** | |  |
| *nrf2* | GCCCTCTTCCGACTGGAATC | CTATGGTGTCCAGGCCTTCC |
| *keap1a* | TGCACCTAACCGTGGAACTC | GGTTCCTGGGGATGTTGAGG |
| *sod* | TGGCAAGAACAAGAACCACA | CCTCTGATTTCTCCTGTCACC |
| *cat* | TCCAGGATGTGGTGTTCACG | AGCTGATCCTGATTCGCCAG |
| *gst* | AACTTTTCGCTGGCTGATGT | TCTTGTCCCTGTGGGTTCTC |
| *gpx* | TCGTTACACTGCCAAGGGAC | TCGTTCCTACAGACGGGACT |
| *il-10* | TCTCCTCCTGTCCGTCCTGGTT | GTAAGCTGTTCATGGCGTGGCA |
| *tgf-β* | GCTCAAAGAGAGCGAGGATG | TCCTCTACCATTCGCAATCC |
| *il-1β* | CCCCAAGATGCCTAAGGGAC | CAGTTGTTCGTCCGTCCTGA |
| *tnf-α* | CAGCACCTCCTCCTTCTCCTCT | TTTGGCACACCGACCTCACC |
| ***Peptide and AA transporters*** | | |
| *slc7a5* | CCAAAGCACGACAGACCTACA | ACCAACCTGGCATATTTCACC |
| *slc7a8* | GGTGACCACAGGGATAGAGATG | TTGCTTACGGAGGCTGGAACTT |
| *slc38a2* | AATAGGGAAAAGCACCACGGG | GTATGAGCAGCTCAAAGACCG |
| *slc15a2* | TGCACATCCCCTCTAGTACG | CAAGTCAGTTGGAGCCATTCC |
| ***GH-IGF system*** | |  |
| *igf1* | CTTCAAGAGTGCGATGTGC | GCCATAGCCTGTTGGTTTACTG |
| *irs1* | TAGTGGTGGTGTCAGCGGT | GGAGGTGGAAGTAAAGGAT |
| *ir* | CATTTTGAGGGAACTGGGTC | CTTGATGATGTCTTTAGCGA |
| *pi3kr1* | AAGACCTTCCTCATCACGAC | CCTTCCACTACAACACTGCA |
| ***TOR signaling pathway*** | |  |
| *s6* | GCCAATCTCAGCGTTCTCAAC | CTGCCTAACATCATCCTCCTT |
| *tor* | TCAGGACCTCTTCTCATTGGC | CCTCTCCCACCATGTTTCTCT |
| *akt1* | CACCGTAGAACCGAGCCCGCT | CGCCATGAAGATCCTAAAGAA |
| *4ebp1* | AGCAGGAAGCCCATCCAAAA | GTCAATGGGCAGTCAGAAGA |
| ***AAR signaling pathway*** | |  |
| *chop* | TGGTGGTGTTGATGGTGGTAA | AGACGTGGGGTGAGGGTGTTC |
| *redd1* | TGACCTGTGTCCCTCTAATGA | ATGTGCTCCAGAAGTTTCTCA |
| *eIF2α* | TAAGTCCAGCCCATCCAAAA | CACCCGAGGAGGCCATCAAG |
| ***Muscle growth and differentiation factors*** | | |
| *mef2a* | TGTCTAACCCCTCCCTCTCTC | CCCTCTGGCTTCCCTCCTCAA |
| *mstn* | GCTCTCCGTCCCAACTCAAG | AAGTACCCGCACACCCACCT |
| *myog* | TTGGGAATGTCCACAGGAAAGG | CACACAGAGCTACAGCAGCGAG |
| *myod* | CCTCACCCTCCCCTCTCTC | TTCACCTTGCTTAGTCGCCG |
| *pax7a* | GCCAAATCACATCCGACACAA | GATCTCCCAGCTGAACATACC |
| *myf5* | CATCTGAATGCAAACTACAGCA | AATATAGCCACGTTAAAGCGAA |
| ***Reference gene*** | | |
| *β-actin* | AAAGGGAAATCGTGCGTGAC | AAGGAAGGCTGGAAGAGGG |
| *EF1α* | TGCTGCTGGTGTGTTGGTGAGTT | TTCTGGCTGTAAGGGGGCTC |
